# The development of nations conditions the disease space

**DOI:** 10.1371/journal.pone.0244843

**Published:** 2021-01-07

**Authors:** Antonios Garas, Sophie Guthmuller, Athanasios Lapatinas

**Affiliations:** 1 Chair of Systems Design, ETH Zurich, Zurich, Switzerland; 2 Joint Research Centre, European Commission, Ispra, VA, Italy; 3 Health Economics and Policy Group, Institute for Social Policy, Department of Socioeconomics, Vienna University of Economics and Business, Vienna, Austria; 4 RWI Research Network, RWI Essen, Essen, Germany; Universidad Rey Juan Carlos, SPAIN

## Abstract

Using the economic complexity methodology on data for disease prevalence in 195 countries during the period of 1990-2016, we propose two new metrics for quantifying the disease space of countries. With these metrics, we analyze the geography of diseases and empirically investigate the effect of economic development on the health complexity of countries. We show that a higher income per capita increases the complexity of countries’ diseases. We also show that complex diseases tend to be non-ubiquitous diseases that are prevalent in disease-diversified (complex) countries, while non-complex diseases tend to be non-ubiquitous diseases that are prevalent in non-diversified (non-complex) countries. Furthermore, we build a disease-level index that links a disease to the average level of GDP per capita of the countries in which the disease is prevalent. With this index, we highlight the link between economic development and the complexity of diseases and illustrate how increases in income per capita are associated with more complex diseases.

## Introduction

There is strong historical evidence that the wealth of nations is positively linked to the health of their populations. Since the eighteenth century, economic development associated with improvements in nutrition, access to sanitation, public health interventions, and medical innovations such as vaccination, have contributed to the reduction of major infectious diseases, the decline of premature death rates, and a longer life expectancy for children and adults in both developed and developing countries [1–4].

Nevertheless, many significant health problems have emerged in concert with economic development and technological modernization. Among them, stress, anxiety, sleep deprivation, and depression are mental disorders that are more prevalent in high-income countries. While they account for only 9% of the burden in low-income countries, this figure is 18% in middle-income and 27% in high-income countries [5]. In OECD countries, a longer life expectancy is coupled with a higher rate of chronic and long-term illnesses in older populations [6]. Industrialization has expanded the reach of existing food-related diseases and created new disorders and addictions [7]. Industrialization also stimulates urbanization, the process of population migration from rural areas to cities. This makes urban areas focal points for many emerging environmental and health hazards [8, 9]. Industrialization is also linked to occupational accidents and work-related diseases (e.g., work-related cancers, musculoskeletal disorders, respiratory diseases, psycho-social problems, and circulatory diseases), which are worldwide problems resulting in important losses for individuals, organizations and societies [10–17]. From the above discussion, it becomes clear that economic development can affect population health in a number of ways, both positive and negative.

To study the determinants of a country’s level of health, and in particular its link with economic development, a number of indicators are routinely used to measure population health, such as mortality, life expectancy or a set of morbidity measures (including incidence, prevalence, and disability adjusted life years). Those indicators share the common aim of summarizing the burden of diseases, and allow cross-country comparison as well as comparison between diverse diseases, health conditions, and risk factors. While there is evidence that the *amount* of health or diseases in a country is linked with its economic development, little is known about the impact of economic development on the disease composition or disease structure of a country.

To disentangle the relationship between economic development and population health and gain more insight on countries’ health level and co-occurence of diseases, we follow a holistic approach and explore (a) the country-disease network, which places countries with similar diseases close together, and (b) the disease space, i.e., the network representation of the co-occurrence of diseases worldwide. In this way, we can capture disparate diseases and health conditions, and their intricate relationships into a unified measure to study its link with economic development.

The relatedness of diseases has been previously studied at the micro level; Goh et al. in 2007 [18], who derive disease networks linking gene co-occurrence in various disorders, and Hidlago et al. in 2009 [19], who use phenotypic connections between diseases to derive a Phenotypic Disease Network (PDN). Similarly, we construct the disease space linking country co-occurrence of diseases. In this setting, explaining why countries are sharing a similar structure of diseases is less straightforward as the underlying causes of co-occurrences of diseases at the country level are multiple. These countries might for instance share the same type of risk factors, environment, population characteristics or lifestyles, and similar health care systems. Thus, when a country is affected by a disease located in the core of the disease space, many other similar countries are also affected by this disease. Studying the structure of the disease space and knowing where a country is located in the country-disease network is therefore a useful tool to monitor the evolution of diseases and plan global health policies.

The country-disease network and the disease space reveal information about the income of populations and their health-related habits, such as lifestyle and dietary habits [20, 21]. There are also multiple reasons to expect countries’ disease structures to be associated with their ‘structural transformations’ (i.e., the industrialization process by which economies diversify from agriculture to manufacturing and services [22–26]), environmental performance [27–30], or health-related policies [31–34], as these contribute to their health status and living standards [35, 36].

We develop a new metric called the *Health Complexity Index*, HCI, that quantifies the disease space by assigning lower values to countries with diseases located at the periphery of the disease space and higher values to countries with diseases located at the centre of the disease space (the diseases themselves are characterized by the corresponding *Disease Complexity Index*, DCI, with lower values assigned to diseases located at the periphery of the disease space and higher values to diseases located at the centre of the disease space). The HCI measures the composition of a country’s pool of diseases by combining information on the complexity of diseases, i.e., the number of countries in which the diseases have prevalent cases (ubiquity) and the disease-diversity of these countries (the number of disease types).

In this view, *disease complexity* is different from a disease requiring a complex treatment or a disease with complex causes i.e. a multi-factorial disease. Thus, in this paper, we use the term *complex disease* to refer solely to the concept of *disease complexity* that measures instead whether a disease is located in the densely connected core of the disease space i.e., whether many other related diseases are present in many other countries.

We show that relatively high scores on the HCI indicate countries with complex diseases. These high-complexity diseases tend to be prevalent in only a few disease-diversified countries, while low HCI scores refer to countries with low-complexity diseases, that are diseases that tend to be prevalent in only a few non-diversified countries.

Moreover, we show that complex countries (diseases) tend to be (prevalent in) richer countries, while countries (diseases) with a low HCI (DCI) tend to be (prevalent in) poorer countries. The descriptive analysis also shows that non-complex diseases tend to be communicable diseases, while complex diseases tend to be non-communicable diseases for which personal lifestyles and societal conditions associated with economic development are important determinants [37–40].

To compute the HCI, we follow the economic complexity methodology, which was initially applied to trade micro-data, measuring the amount of knowledge materialized in a country’s productive structure [41]. Based on the ECI methodology, a number of recent contributions explain economic development and growth as a process of information development and of learning how to produce and export more diversified products [41–55]. Furthermore, [56] have recently shown that countries exporting complex products tend to be more inclusive and have lower levels of income inequality than countries exporting simpler products. In addition, [57] find that countries with high intellectual quotient (IQ) populations produce and export more sophisticated/complex products, while [58] shows that the Internet has a positive effect on economic complexity. Adopting the economic complexity methodology, [59] compute a knowledge complexity index with more than two million patent records for US metropolitan areas between 1975-2010. They analyze the geography and evolution of knowledge complexity in US cities and show that the most complex cities in terms of patents are not always those with the highest rates of patenting. In addition, using citation data, they show that more complex patents are less likely to be cited than simpler patents when the citing and cited patents are located in different metropolitan areas.

The aim of this paper is fourfold: (*i*) to build two new metrics that quantify the disease space, following the economic complexity methodology; (*ii*) to explore the link between countries’ health complexity and their economic performance using the new metrics and following dynamic panel data analysis; (*iii*) to develop a disease-level index that links a disease to the average level of *GDP* *per* *capita* of the countries in which the disease is prevalent; (*iv*) to illustrate how a country’s economic development is associated with changes in its disease composition and verify the relationship between economic development and health complexity at the disease level.

The remainder of the paper is structured as follows. Section ‘The country-disease network’ describes the data on disease prevalence and the construction of the country-disease network. Section ‘Methods’ presents the methodology for constructing the disease space and computing the HCI and the DCI. Section ‘The geography of complex diseases’ presents the results of the structural analysis of the disease space, with a particular focus on the geography and evolution of health complexity across countries and regions. Section ‘The effect of economic development on health complexity’ empirically investigates the relationship between economic development and health complexity using the HCI, data on *GDP* *per* *capita*, and potential covariates. Section ‘Economic development and disease complexity’ introduces an index that decomposes economic performance at the disease level. Finally, in Section ‘Conclusions’, we offer some concluding remarks.

## The country-disease network

### Data on diseases and injuries

Information on diseases and injuries comes from the Global Burden of Diseases (GBD) study led by the Institute for Health Metrics and Evaluation (IHME), an independent population health research centre at the University of Washington (UW Medicine) [60]. The GBD study has an established reputation for providing comparable estimates of the prevalence of diseases and injuries across the world [61].

We used the 2016 version of the GBD study, which collects and analyzes a continuous stream of the most up-to-date data available from a wide variety of sources (administrative records, randomized controlled trials, cohort studies, surveys, census, scientific literature, satellite data, vital registration data) [62]. The dataset covers more than 300 diseases and injuries for 195 countries in the period of 1990-2016. S1 and S2 Tables list the countries and diseases/injuries included in our dataset. The data are extracted into centralized databases to model internally consistent estimates for diseases, injuries, and risk factors, adjusting for bias, standardizing for age and incorporating covariates [61, 63]. GBD data and their descriptive analysis are publicly available through interactive tools and visualizations (https://vizhub.healthdata.org/gbd-compare/). The development of GBD methods/estimates, including statistical techniques for missing data, are discussed in [62, 64–66]. The GBD methodology is reviewed and updated annually and is compliant with the Guidelines for Accurate and Transparent Health Estimates Reporting (GATHER) [67].

As described in the methodological appendix of the GBD 2016 study (S1 Appendix), input data and data sources are less detailed and numerous in some low-income countries, compared to developed countries; however, GBD 2016 estimates take into account under- and/or miss-reporting of data, adopting up-to-date error correction methods that improve the reliability of the recent estimates.

We acknowledge that differences in the accuracy of disease classifications between developed and developing countries might influence the disease space, but as [68] show, this effect probably explains an insignificant part of our results. The authors find that the incidence rate of all-sites cancer increases with per capita income, even after controlling for improvement in cancer detection. In other words, if higher incidence rates in developed countries were merely due to improved cancer detection, the authors would have found a flat or even an inverted-U relationship between per capita income and cancer incidence.

Diseases and injuries are grouped by causes. The broader classification of causes (level 1) includes: (a) *communicable, maternal, neonatal, and nutritional diseases* such as HIV/AIDS and sexually transmitted infections, respiratory infections and tuberculosis, enteric infections (e.g., diarrheal diseases, typhoid fever), neglected tropical diseases (e.g. malaria, chagas disease) and other infectious diseases (e.g. meningitis and acute hepatitis), maternal and neonatal disorders (e.g., maternal abortion and miscarriage, ectopic pregnancy, maternal obstructed labor and uterine rupture), nutritional deficiencies (e.g., protein-energy malnutrition, vitamin A, iron, iodine deficiencies); (b) *non-communicable diseases* such as cancers, cardiovascular diseases, chronic respiratory diseases, digestive diseases (e.g., cirrhosis, gastritis, pancreatitis), neurological disorders (e.g., multiple sclerosis, epilepsy, Parkinson’s and Alzheimer’s diseases, migraine), mental disorders (e.g., schizophrenia, anorexia nervosa and bulimia nervosa, conduct and hyperactivity disorders), substance use disorders (e.g., alcohol and drug use disorders), diabetes, kidney diseases, skin diseases (e.g., dermatitis, bacterial skin diseases), sense organ diseases (e.g., glaucoma, cataract, vision loss), musculoskeletal disorders (e.g., osteoarthritis, rheumatoid arthritis); (c) *injuries* such as transport injuries (e.g., pedestrian road injuries, cyclist and motorcyclist road injuries), unintentional injuries (e.g., falls, poisonings, exposure to mechanical forces), self-harm and interpersonal violence (e.g., sexual violence, conflict and terrorism, executions). In the remainder of the paper we use the word ‘disease’ to refer to all diseases and injuries classified in the GBD study.

We use information for the most detailed level of causes in the GBD taxonomy (i.e., level 4, and when there is no level 4 classification, we use level 3). For example, among the non-communicable diseases (level 1), neoplasms (level 2) include the following level 3 categories: lip and oral cavity cancer, nasopharynx cancer, other pharynx cancer, esophageal cancer, stomach cancer, colon and rectal cancer, liver cancer, gallbladder and biliary tract cancer, pancreatic cancer, larynx cancer, etc. Then, liver cancer includes the following level 4 subcategories: liver cancer due to hepatitis B, liver cancer due to hepatitis C, liver cancer due to alcohol use, liver cancer due to non-alcoholic steatohepatitis (NASH), liver cancer due to other causes.

Two measures of disease prevalence are exploited: the rate of prevalence (number of cases per 100,000 population) for all ages, and the age-standardized rate of prevalence to account for the differences in age structures across countries. This is useful because relative over- or under-representation of different age groups can obscure comparisons of age-dependent diseases (e.g., ischemic heart disease or malaria) across populations.

### The country-disease bipartite network

Instrumental to our analysis is the bipartite network mapping of countries and diseases. Bipartite, or bi-modal networks are abundant in the scientific literature, with examples including the city-tech knowledge network [59], the city-firm network [69], firm-projects networks [70], predator-prey networks [71], plants-pollinator networks [72] etc. Here, we use data from the 2016 Global Burden of Diseases study that assessed the disease burden of countries in the period of 1990 to 2016, and we generate an *l* × *k* country-diseases matrix **E**, were the matrix element *E*_*cd*_ represents the number of cases of disease *d* per 100,000 population in country *c*.

The aforementioned matrix allows for the construction of an undirected, weighted country-disease network by linking each disease to the countries with disease cases. These networks are very dense, and in order to visually explore their structure, we apply the Dijkstra algorithm [73] to extract a Maximum Spanning Tree (MST) that summarizes their structures. More precisely, the MST, which is usually considered as the backbone of the network, is a connected subgraph having *l* + *k* − 1 edges with the maximum total weight and without forming any loops.

**Fig 1:**
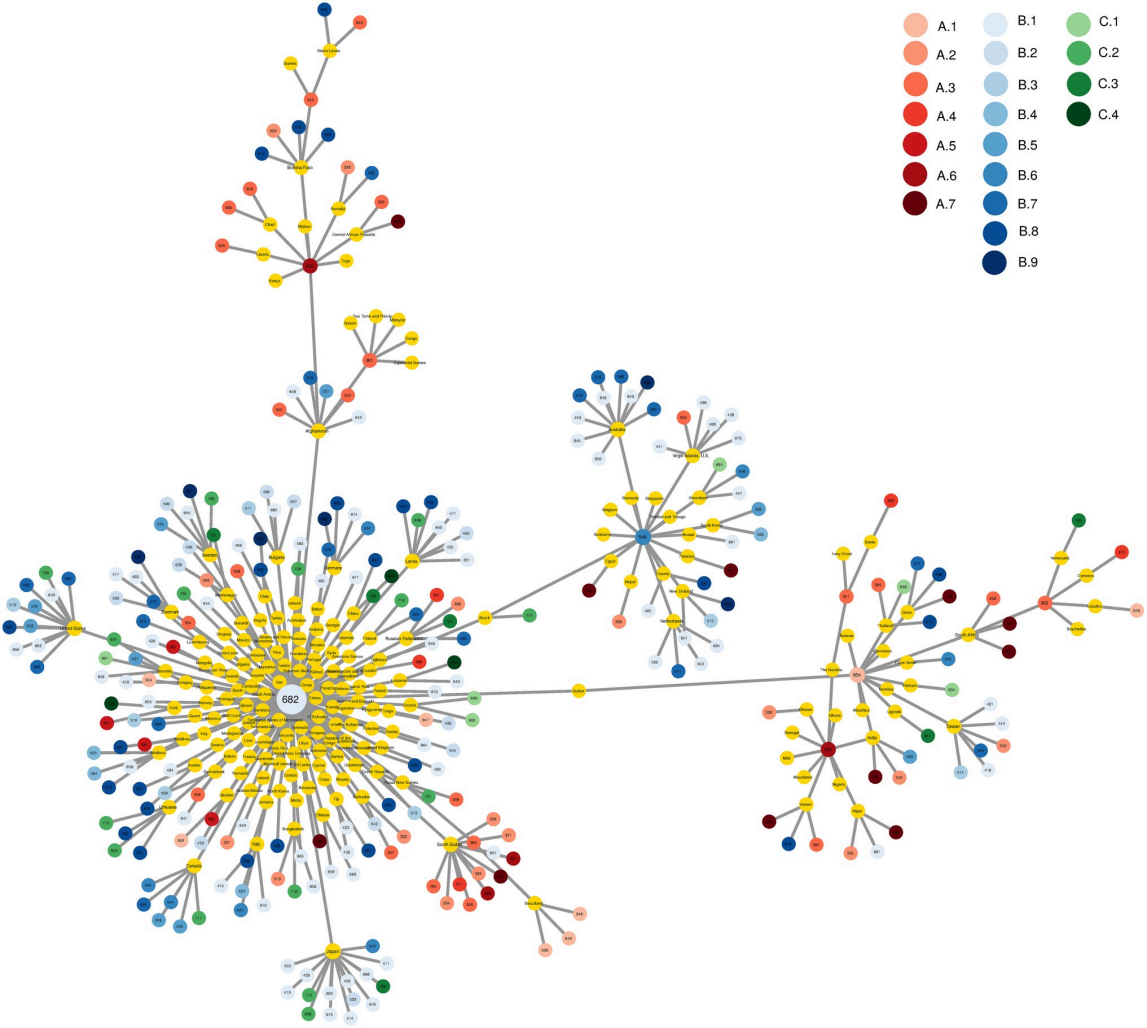
Maximum spanning tree of the country-disease bipartite network. Countries are represented by yellow nodes, and diseases cover the following categories: [A. ‘Communicable, maternal, neonatal, and nutritional diseases’] A.1 ‘HIV/AIDS and tuberculosis’, A.2 ‘Diarrhea, lower respiratory, and other common infectious diseases’, A.3 ‘Neglected tropical diseases and malaria’, A.4 ‘Maternal disorders’, A.5 ‘Neonatal disorders’, A.6 ‘Nutritional deficiencies’, A.7 ‘Other communicable, maternal, neonatal, and nutritional diseases’; [B. ‘Non-communicable diseases’] B.1 ‘Neoplasms’, B.2 ‘Cardiovascular diseases’, B.3 ‘Chronic respiratory diseases’, B.4 ‘Cirrhosis and other chronic liver diseases’, B.5 ‘Digestive diseases’, B.6 ‘Neurological disorders’, B.7 ‘Mental and substance use disorders’, B.8 ‘Diabetes, urogenital, blood, and endocrine diseases’, B.9 ‘Musculoskeletal disorders’; [C. ‘Injuries’] C.1 ‘Transport injuries’, C.2 ‘Unintentional injuries’, C.3 ‘Self-harm and interpersonal violence’, C.4 ‘Forces of nature, conflict and terrorism, and executions and police conflict’. Data for 2016.

In Fig 1 we illustrate the country-disease MST for 2016. From this MST, we can easily identify clusters of countries that are linked to specific types of diseases. The main node of the network is caries in permanent teeth (disease cause number 682; see S2 Table for the list of diseases). This disease is the most common disease across the world, as it is present in the majority of countries. It is also the disease with the highest prevalence worldwide (2.44 billion cases in 2016 [60]).

## Methods

To calculate *health complexity*, we combine information on diseases prevalence and how common these diseases are across countries, following the economic complexity methodology, i.e., the formulas in the pioneering work of [49].

### The relative disease disadvantage

In short, let us assume that we have disease information for *l* number of countries and *k* diseases. With this information, we can fill an *l* × *k* diseases matrix **E**, so that matrix element *E*_*cd*_ is country *c*’s information for disease *d*. If there is no information for disease *d* in country *c*, then *E*_*cd*_ = 0. From this matrix, it is easy to calculate the following ratio:

$$\begin{array}{c} {\text{RDD}}_{cd}=\frac{X_{cd}\sum_{d^{\prime }}X_{cd^{\prime }}}{\sum_{c^{\prime }}X_{c^{\prime }d}\sum_{c^{\prime }d^{\prime }}X_{c^{\prime }d^{\prime }}}, \end{array}$$ (1)

where *X*_*cd*_ is the number of cases of disease *d* per 100,000 population in country *c*.

Similar to the economic complexity methodology and the discussion in [44, 49, 56], we claim that a country has a relative disease disadvantage in a disease when RDD_cd_ ≥ 1. In other words, a country *c* has an RDD in disease *d* if the proportion of its cases in the country’s pool of all disease cases is higher than the proportion of disease *d*’s cases in the world’s pool of all disease cases.

**Fig 2:**
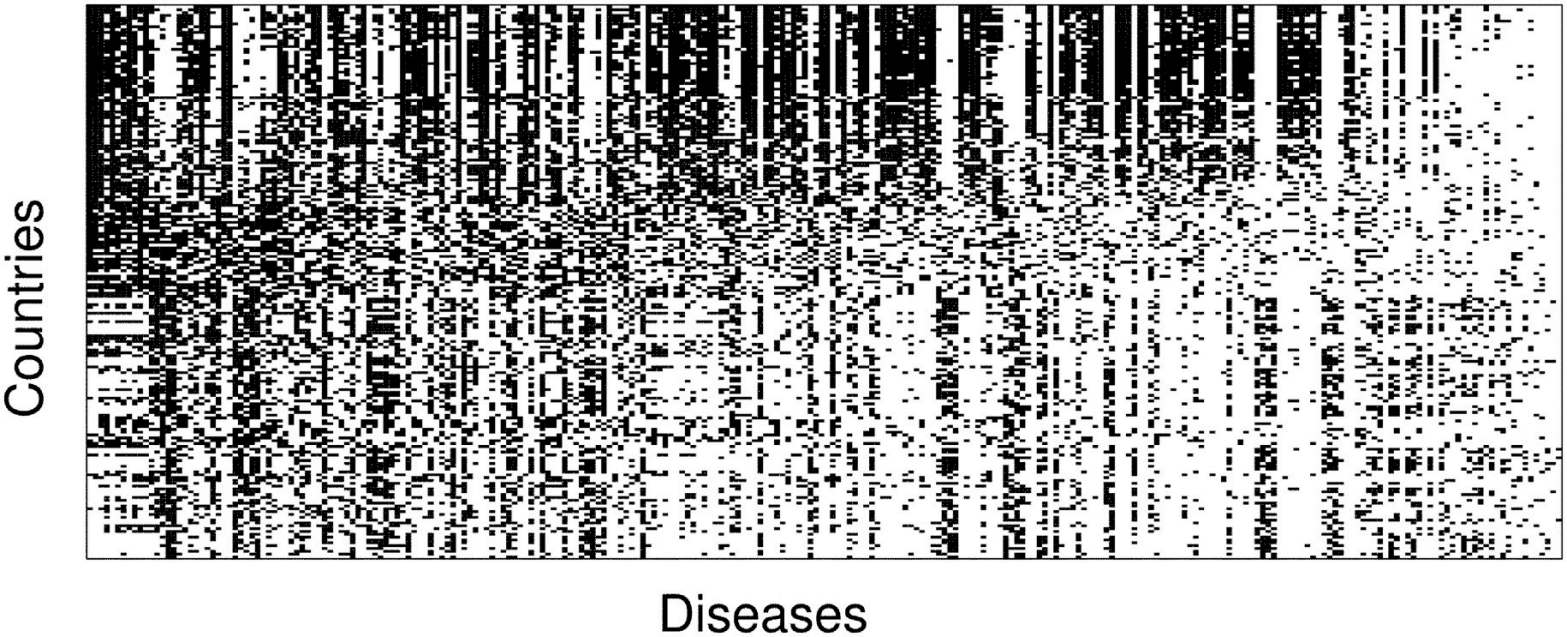
Matrix representation of the links between countries and diseases. A visualization of this matrix for the year 2016, where a dark point indicates that country *c* has an RDD in a given disease *d*. The matrix is sorted by row and column sums. While the existence of countries that are very well diversified and countries that have a relative disease disadvantage only in a small set of diseases is clear, we would like to note that contrary to applications of this methodology to country-export data, for the disease prevalence this matrix is not clearly triangular (or nested).

Using this threshold value, we obtain the *l* × *k* matrix **M**, with matrix elements *M*_*cd*_ = 1 if country *c* has an RDD in disease *d*, and zero otherwise. A visualization of the matrix **M** for this dataset is shown in Fig 2, where a dark point indicates that country *c* has an RDD in a given disease *d*. The matrix is sorted using the NODF algorithm [74], which highlights the existence of countries that are very well diversified and countries that have an RDD only in a small set of diseases.

In what follows, we assume that a disease *d* is prevalent in country *c* when RDD_cd_ ≥ 1.

### The disease space

The clustering of countries and diseases in the MST of the country-disease network already points towards relations in the prevalence of different diseases (Fig 1). To explore this further, we construct the disease space, similar to the product-space introduced by [41].

**Fig 3:**
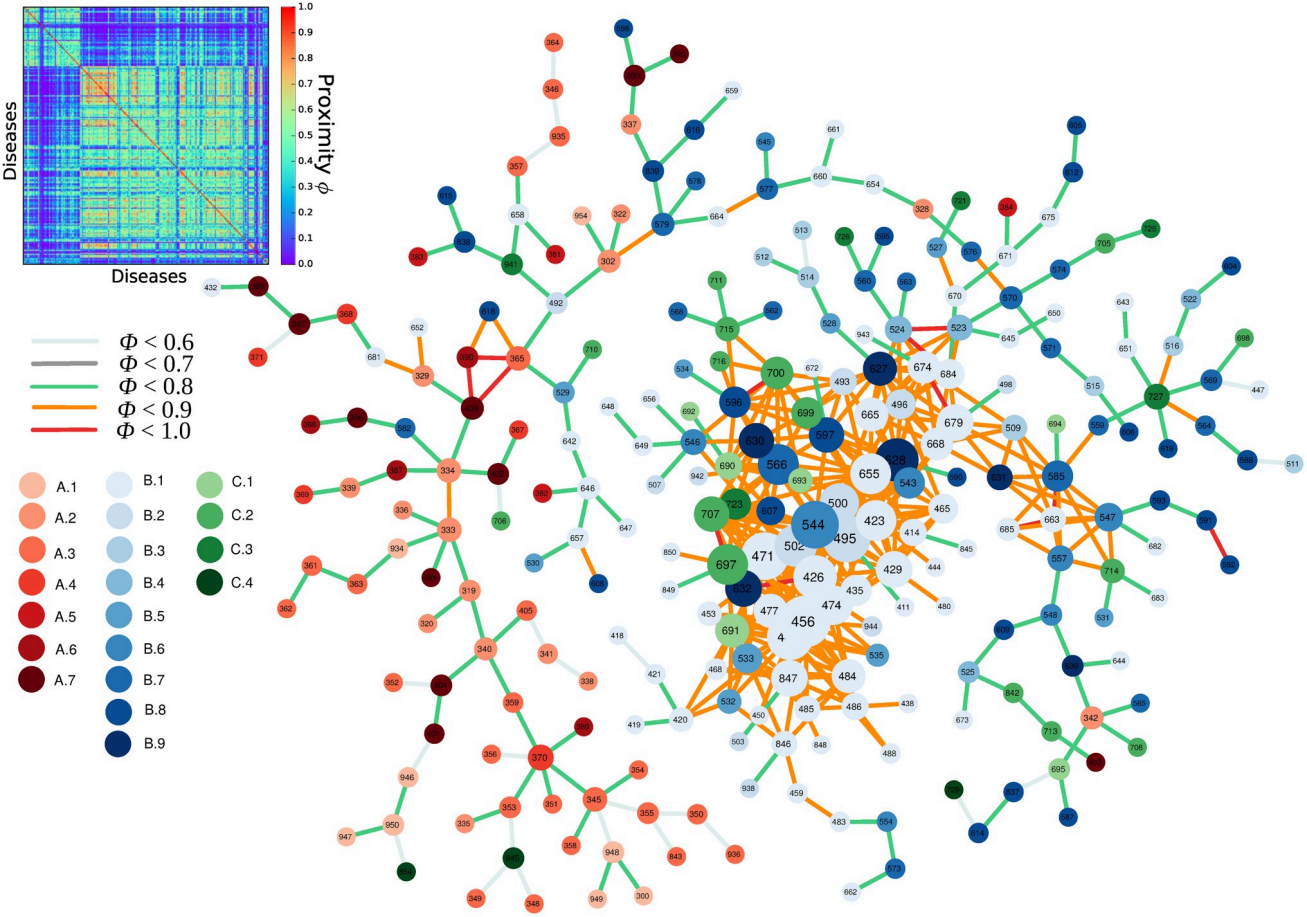
The proximity matrix and the resulting disease space. The size of the nodes is proportional to their degree, i.e., the number of links. Disease colors cover the following categories: [A. ‘Communicable, maternal, neonatal, and nutritional diseases’] A.1 ‘HIV/AIDS and tuberculosis’, A.2 ‘Diarrhea, lower respiratory, and other common infectious diseases’, A.3 ‘Neglected tropical diseases and malaria’, A.4 ‘Maternal disorders’, A.5 ‘Neonatal disorders’, A.6 ‘Nutritional deficiencies’, A.7 ‘Other communicable, maternal, neonatal, and nutritional diseases’; [B. ‘Non-communicable diseases’] B.1 ‘Neoplasms’, B.2 ‘Cardiovascular diseases’, B.3 ‘Chronic respiratory diseases’, B.4 ‘Cirrhosis and other chronic liver diseases’, B.5 ‘Digestive diseases’, B.6 ‘Neurological disorders’, B.7 ‘Mental and substance use disorders’, B.8 ‘Diabetes, urogenital, blood, and endocrine diseases’, B.9 ‘Musculoskeletal disorders’; [C. ‘Injuries’] C.1 ‘Transport injuries’, C.2 ‘Unintentional injuries’, C.3 ‘Self-harm and interpersonal violence’, C.4 ‘Forces of nature, conflict and terrorism, and executions and police conflict’. Data for 2016.

Calculating the RDD for all country-disease pairs allows us to derive a matrix **Φ**, whose elements *Φ*_*i*,*j*_ define a proximity measure between all pairs of diseases. This proximity measure reveals diseases that are present in tandem, or in other words, with **Φ**, we measure the probability that a country *c*, which has a relative disease disadvantage in disease *i*, also has a relative disease disadvantage in disease *j*. The proximity measure is defined as:

$$\begin{array}{c} \Phi_{i,j}=\text{min}\left\{\text{Pr}\left({\text{RDD}}_{i}\geq 1\;|\;{\text{RDD}}_{j}\geq 1\right),\;\text{Pr}\left({\text{RDD}}_{j}\geq 1\;|\;{\text{RDD}}_{i}\geq 1\right)\right\}, \end{array}$$ (2)

where Pr(RDD_*i*_ ≥ 1 | *RDD*_*j*_ ≥ 1) is the conditional probability of having a relative disease disadvantage in disease *i* if you have a relative disease disadvantage in disease *j*. Using the minimum of both conditional probabilities, we avoid issues of a rare disease being present in only one country. Additionally, we make the resulting matrix **Φ** symmetric (see Fig 3). The proximity matrix is highly modular and its block structure reveals the presence of ‘communities’, i.e., groups of diseases that are expected to occur together.

Next, we map this matrix onto a network, where each disease is represented by a node and every matrix element represents a weighted and undirected link. Similar to Fig 1, we start by applying Dijkstra’s algorithm on matrix **Φ** which calculates the MST of the network. Following the rationale of [41], we start from the strongest links that are not part of the MST and keep adding links to the network until the average degree is four. The resulting network is a visual representation of the disease space, which is shown in Fig 3.

From Fig 3, it is evident that in the disease space network, different disease categories are clustered together and the network is heterogeneous and follows a core-periphery structure. The external part of the network (the periphery) is mostly dominated by ‘communicable, maternal, neonatal, and nutritional diseases’. On the other hand, the core of the network is dominated by ‘non-communicable diseases’.

As reported by [19], disease prevalence, i.e., the number of times a particular disease is diagnosed, follows a heavy-tailed distribution. In this case, the economic complexity methodology becomes a handy tool as it allows us to link a country to a particular disease only if it has a relative disease disadvantage. As was shown by [75], economic-complexity-based indices are related to various dimensionality-reduction methods and are equivalent to a spectral clustering algorithm. This allows the indices to be interpreted as vectors that determine distances between nodes based on their similarity, which is a nice way to interpret the disease-space network. In our case, we reduce the space of the various ways that diseases could be linked together, and we are only interested in identifying links between diseases with a large probability of co-occurrence.

We highlight that disease prevalence is starkly different to product exports that are used in applications of the economic complexity methodology. Countries acquire capabilities over time that enable them to develop more sophisticated products and increase the complexity of their production. This process is a cumulative one, i.e., countries are constantly increasing their product portfolio by developing new capabilities without loosing previously acquired ones. In contrast, as countries become more developed, they are more effective at fighting diseases, e.g., by using new treatments or vaccines and by improving the general hygiene of the population. Therefore, the set of diseases in a country does not increase monotonically in time, as new diseases are added and some diseases disappear. Thus, we expect our results to be somehow different to findings based on export data, and the first hint is already provided by the structure of Fig 2, which is less nested compared to similar figures in other applications of economic complexity.

### Health complexity index

From matrix **M**, similar to [49], we introduce the HCI as a measure of countries’ disease structures. To obtain the HCI, we first calculate the *l* × *l* square matrix $\tilde{M}$. In short, matrix $\tilde{M}$ provides information about links connecting two countries *c* and *c*′, based on the disease cases in both. The matrix elements ${\tilde{M}}_{cc^{\prime }}$ are computed as

$$\begin{array}{c} {\tilde{M}}_{cc^{\prime }}=\frac{1}{k_{c,0}}\sum_{d}\frac{M_{cd}M_{c^{\prime }d}}{k_{d,0}}, \end{array}$$ (3)

where *k*_*c*,0_ = ∑_*d*_ *M*_*cd*_ measures the diversification of country *c* in terms of its different diseases i.e. the number of disease-types *d* for which a country *c* has an RDD, and *k*_*d*,0_ = ∑_*c*_ *M*_*cd*_ measures the number of countries with an RDD in disease *d* i.e the ubiquity of disease *d*. If **K** is the eigenvector of $\tilde{M}$ associated with the second largest eigenvalue, then according to [48], the HCI is calculated as

$$\begin{array}{c} \text{HCI}=\frac{K-\langle K\rangle }{std(K)}. \end{array}$$ (4)

As discussed in sub-section ‘The relative disease disadvantage’, the HCI is computed using in *X*_*cd*_ the number of disease cases (according to cause levels 3 or 4) per 100,000 population for 195 countries and for 196 diseases. The time-period covered is from 1990 to 2016. With the age-standardized data (see the discussion in subsection ‘Data on diseases and injuries’, we also calculate the age-standardized health complexity index (AHCI) following the same formulas. We use the two indices as alternative measures when checking the robustness of our results. It should be noted here that the computation of the indices is based only on diseases for which a country has an RDD in terms of disease prevalence (the incidence matrix of the bipartite network linking countries to diseases, **M**, reflects whether or not a country has an RDD in a specific disease; see Fig 2).

### The disease complexity index

In a similar manner, but placing the spotlight on diseases rather than countries, we calculate the *Disease Complexity Index* (DCI) in which the *k* × *k* matrix $\tilde{M}$ provides information about links connecting two diseases *d* and *d*′, based on the number of countries in which both diseases have cases. The DCI is computed using in *X*_*cd*_ the number of disease cases (according to cause levels 3 or 4) per 100,000 population for 195 countries and for 196 diseases. The time-period covered is from 1990 to 2016. With the age-standardized data (see the discussion in subsection ‘Data on diseases and injuries’), we also calculate the age-standardized disease complexity index (ADCI) following the same formulas. Therefore, the matrix elements ${\tilde{M}}_{dd^{\prime }}$ are computed as

$$\begin{array}{c} {\tilde{M}}_{dd^{\prime }}=\frac{1}{k_{d,0}}\sum_{c}\frac{M_{cd}M_{cd^{\prime }}}{k_{c,0}}, \end{array}$$ (5)

and if **Q** is the eigenvector of $\tilde{M}$ associated with the second largest eigenvalue,

$$\begin{array}{c} \text{DCI}=\frac{Q-\langle Q\rangle }{std(Q)}. \end{array}$$ (6)

Table 1 lists the five diseases with the highest and lowest DCI scores averaged over the period of 1990-2016. Interestingly, we see that high-complexity diseases i.e diseases with the highest DCI belong to disease sections that include multi-factorial diseases.

**Table 1:** List of the five diseases with the highest and lowest DCI values during the period of 1990-2016.

Code	Disease name	Disease section	DCI
*Highest DCI*			
459	Malignant skin melanoma	Neoplasms	1.210
441	Colon and rectal cancer	Neoplasms	1.170
502	Peripheral artery disease	Cardiovascular diseases	1.169
456	Pancreatic cancer	Neoplasms	1.159
533	Vascular intestinal disorders	Digestive diseases	1.152
*Lowest DCI*			
345	Malaria	Neglected tropical diseases and malaria	-2.045
350	African trypanosomiasis	Neglected tropical diseases and malaria	-1.978
370	Maternal obstructed labor and uterine rupture	Maternal and neonatal disorders	-1.957
358	Yellow fever	Neglected tropical diseases and malaria	-1.893
340	Tetanus	Other infectious diseases	-1.883

Notes: DCI: Disease Complexity Index; Average values for 1990-2016.

The computation of the indexes was performed using R version 4.0.0.

## The geography of complex diseases

### Ubiquity, diversity, and the HCI

**Fig 4:**
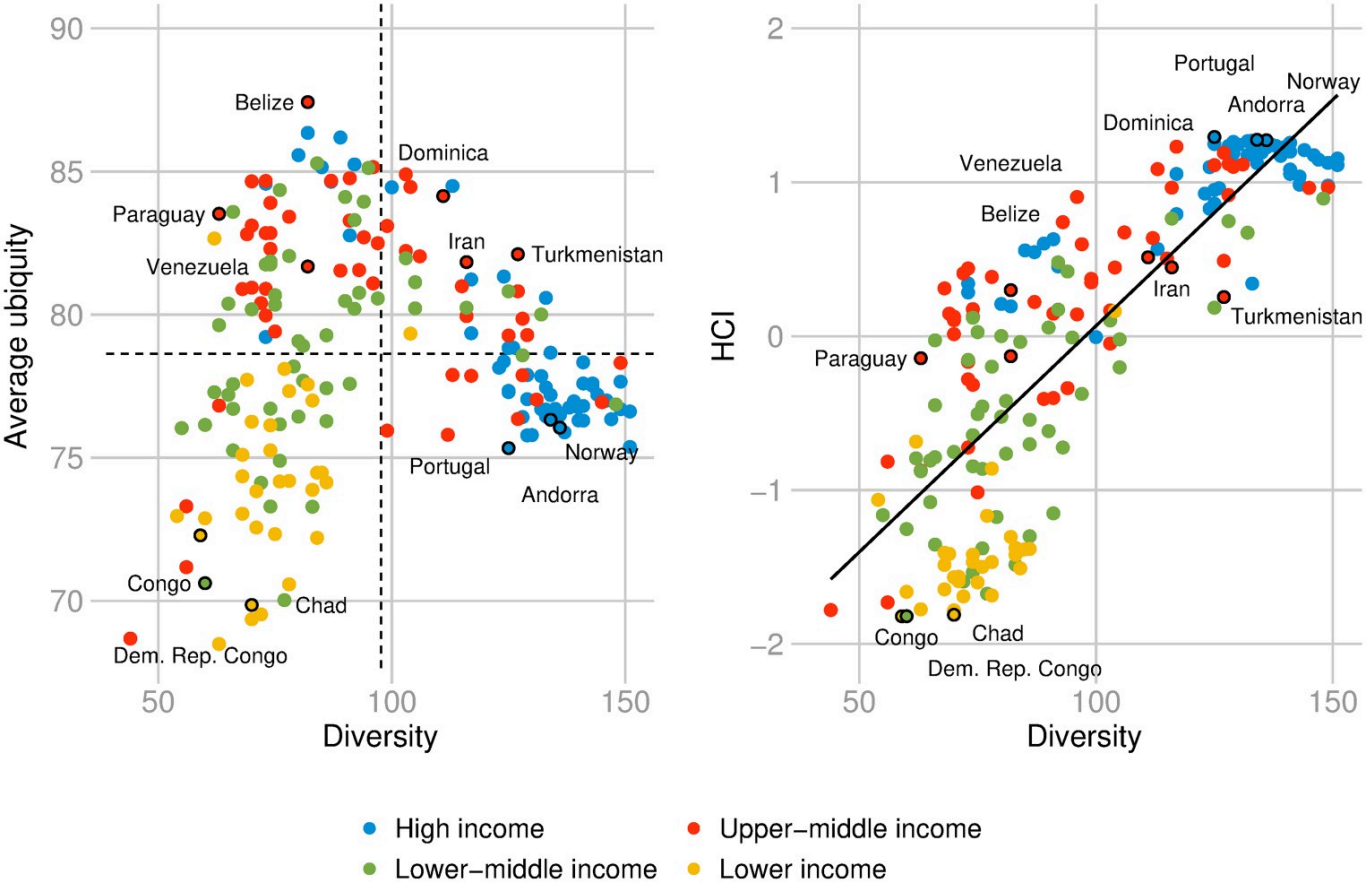
Average ubiquity, diversity, and the HCI. Left panel: Relationship between diversity and average ubiquity. The diagram is divided into 4 quadrants defined by the empirical observed averages of diversity and average ubiquity; Right panel: Relationship between diversity and HCI. The colors depict different income-regions of the world according to World Bank’s classification. Data for 2016.

**Fig 5:**
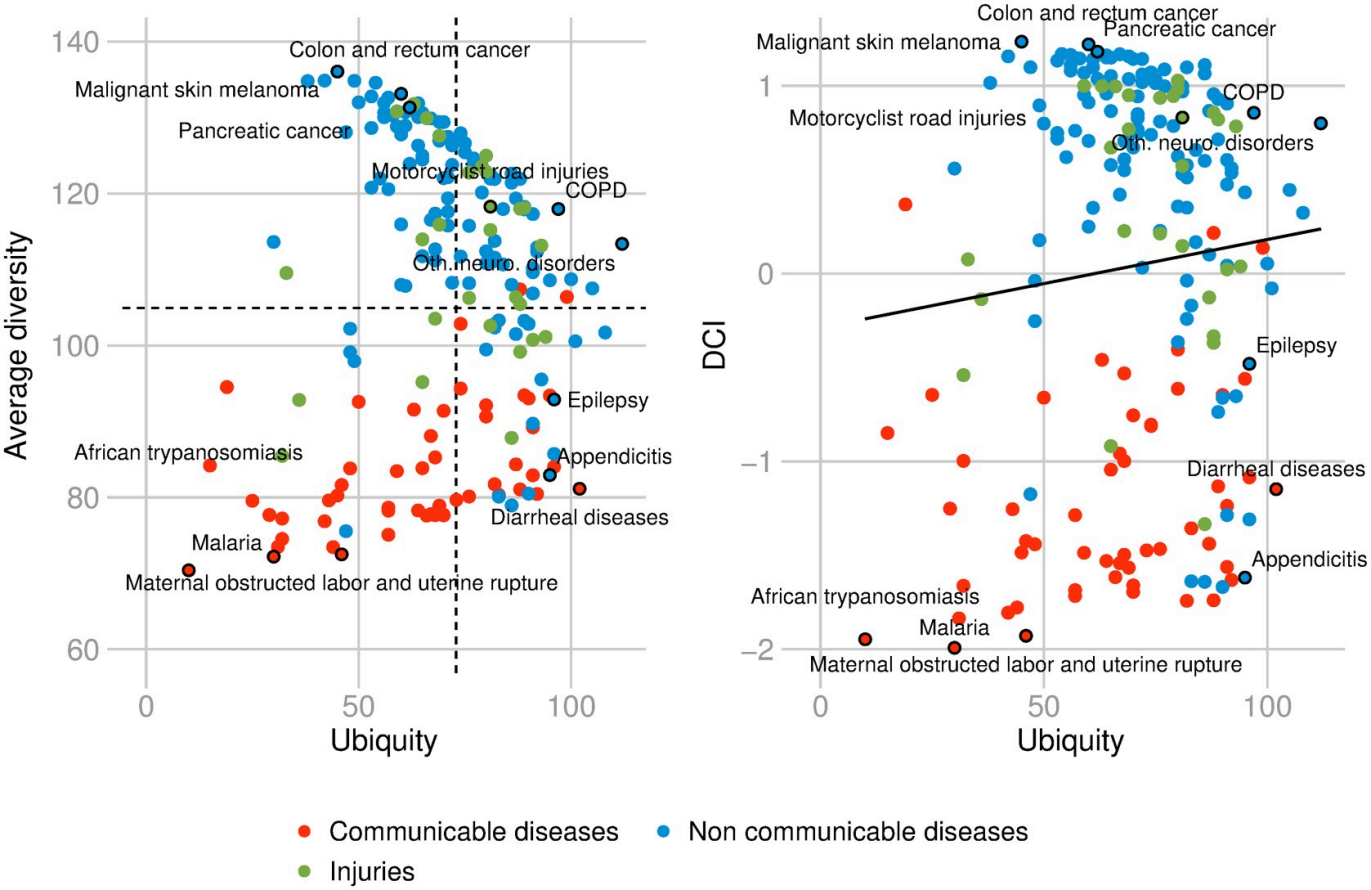
Ubiquity, average diversity, and the DCI. Relationship between ubiquity and average diversity (left). Relationship between average diversity and DCI. The colors depict different disease types. Data for 2016.

In this subsection, we elaborate on the association of the HCI with the diversification of countries and ubiquity of diseases. Figs 4 and 5 provide additional information on where countries/diseases are located in the disease space. From these figures, we can better understand the type of health-related information captured by the economic complexity methodology, and we can explore the way in which the economic complexity methodology can be interpreted as a spectral clustering algorithm when applied to disease data [75, 76].

In Fig 4, the left panel shows the relationship between diversity and average ubiquity of countries in terms of diseases. The different colors represent different income regions of the world according to World Bank’s classification. The diagram is divided into 4 quadrants defined by the empirical observed averages of diversity and average ubiquity. The upper left quadrant refers to non-diversified countries with RDD in ubiquitous diseases. The upper right quadrant depicts the diversified countries with RDD in ubiquitous diseases. The lower left quadrant depicts non-diversified countries with RDD in non-ubiquitous diseases. The lower right quadrant refers to the diversified countries with RDD in non-ubiquitous diseases.

The right panel in Fig 4 depicts a strong positive relationship between diversity and the HCI (*ρ* = 0.8271 with a p-value < 10^−48^). High-HCI countries have many diseases with RDD that tend to be prevalent in only few other disease-diversified countries. Portugal, Andorra, and Norway are some examples of health-complex countries. On the other hand, low-HCI countries have few diseases with RDD, which tend to be prevalent in only few other non-diversified countries, e.g., Congo, Democratic Republic of the Congo, and Chad.

Fig 5 places the spotlight on diseases. The left graph shows the relationship between ubiquity and average diversity of diseases. The right graph shows the relationship between DCI and ubiquity. The two variables are not correlated: *ρ* = 0.0883 with a p-value = 0.2184. This implies that ubiquity fails to be health-informative. The information provided by the two graphs suggests that high-DCI diseases tend to be prevalent in only few disease-diversified countries. These diseases are mostly non-communicable diseases such as ‘colon and rectum cancer’, ‘malignant skin melanoma’, and ‘pancreatic cancer’ (such diseases are located in the upper left quadrant, left graph of Fig 5). On the other hand, low-DCI diseases tend to be prevalent in only few non-diversified countries. These diseases are located in the lower left quadrant. Some examples are ‘malaria’, ‘African typanosomiasis’, ‘maternal obstructed labor and utirine rupture’, and they are typically communicable diseases. Diseases i.e with ubiquity above the average, include, on one side, ‘diarrheal diseases’, ‘appendicitis’, and ‘epilepsy’, which are more present in non-diversified countries, and on the other side, ‘motorcyclist road injuries’, ‘chronic obstructive pulmonary disease (COPD)’ or ‘other neurological disorders’ which tend to be more prevalent in diversified countries (See respectively lower and upper right quadrants, left graph of Fig 5).

**Fig 6:**
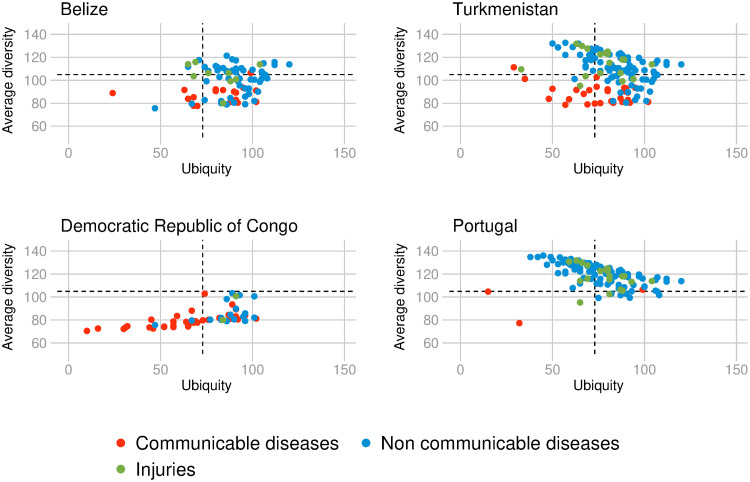
Ubiquity and average diversity of diseases in four selected countries. Relationship between ubiquity and average diversity of diseases for which the country (respectively Belize, Turkmenistan, the Democratic Republic of Congo and Portugal) has a Relative Disease Disadvantage (RDD). The colors depict different disease types. Data for 2016.

The above descriptive findings imply that the HCI places countries on a one-dimensional interval such that countries with many diseases (diversified countries) are close together and countries with only few diseases (non-diversified countries) are far apart. Furthermore, the (non-)diversified countries that tend to have non-ubiquitous diseases are (less) more health-complex compared to the other (non-)diversified countries. For illustrative purposes Fig 6 displays the relationship between the average diversity and ubiquity of diseases for four selected countries. The pool of diseases with RDD in Portugal, the country with the highest HCI in 2016, is composed of many diseases (125) that are prevalent almost exclusively in diversified countries. These diseases are non-communicable diseases (80.80%) and injuries (16%), and include the sixteen highest DCI diseases in 2016: ‘malignant skin melanoma, pancreatic cancer’, ‘colon and rectum cancer’, ‘vascular intestinal disorders’, ‘peripheral artery disease’, ‘gallbladder and biliary tract cancer’, ‘kidney cancer’, ‘Parkinson disease’, ‘mesothelioma’, ‘bladder cancer’, ‘tracheal, bronchus, and lung cancer’, ‘Hodgkin lymphoma’, ‘multiple myeloma, ischemic stroke’, ‘atrial fibrillation and flutter’, ‘brain and nervous system cancer’. Portugal has a RDD in only four communicable disease-types: ‘food-borne trematodiases’, ‘varicella and herpes zoster’, ‘upper respiratory infections’, and ‘HIV/AIDS resulting in other diseases’. On the contrary, in the Democratic Republic of Congo, the country with the lowest HCI in 2016, two-thirds of the diseases are communicable, maternal, neonatal, or nutritional diseases amongst the total of 57 diseases for which the country has an RDD. Those 57 diseases are non-ubiquitous diseases and are only prevalent in other non-diversified countries. In comparison, Belize, located in the upper left quadrant of Fig 4, counts 82 disease-types with RDD. These diseases, of which 65% are non-communicable diseases and 11% injuries, are mostly ubiquitous and prevalent in other non-diversified countries. While for diversified countries with RDD in ubiquitous diseases (situated in the upper right quadrant of Fig 4), such as Turkmenistan, the picture is the following; more than three-quarters of its 127 diseases with RDD, are ubiquitous and non-communicable diseases, with the majority of those, being prevalent in other diversified countries.

**Fig 7:**
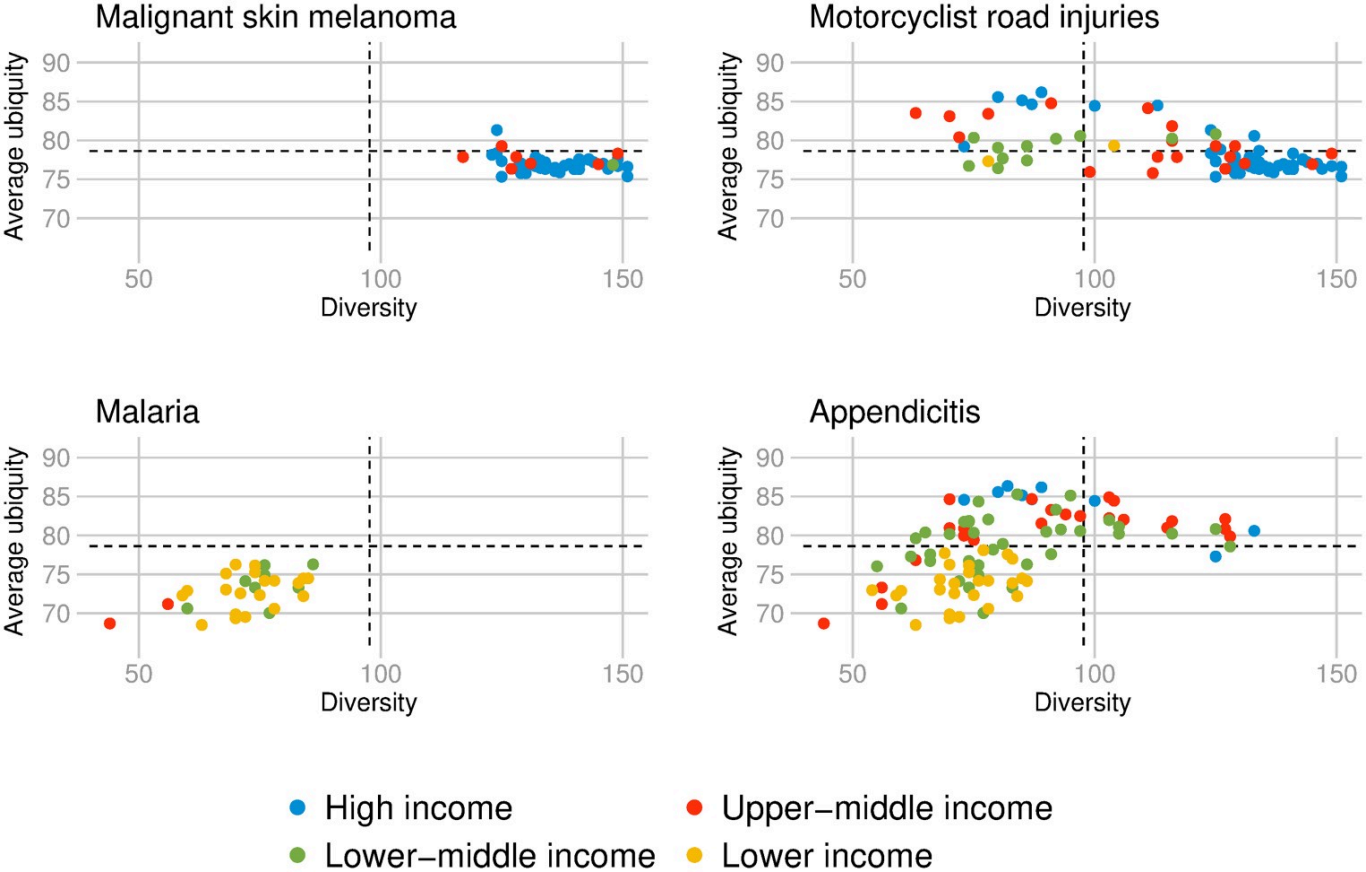
Average ubiquity and diversity of countries in four selected diseases. Relationship between average ubiquity and diversity of countries with RDD in respectively; malignant skin melanoma, motorcyclist road injuries, malaria, and appendicitis. The colors depict different income-regions of the world according to World Bank’s classification. Data for 2016.

For diseases, the dimensionality reduction perspective of the DCI places diseases that are prevalent in more diversified countries close together and diseases that are prevalent in non-diversified countries far apart. Moreover, the (non-)ubiquitous diseases in the former set of diseases are relatively (more) less complex, while the (non-)ubiquitous diseases in the latter set of diseases are relatively (less) more complex. Fig 7 presents the relationship between the average ubiquity and diversity for four selected diseases. ‘Malignant skin melanoma’ is the most complex disease in 2016. As we can see in the upper left graph, the disease appears with an RDD almost exclusively in highly diversified countries that have an RDD in more complex diseases. Those 45 countries are mostly European countries with the highest HCI in 2016, and other high-income countries including the United States, Canada, New Zealand, and Australia. In contrast, ‘malaria’ is exclusively prevalent in 30 non-diversified countries that have an RDD in non-complex diseases. Examples include the sixteen less-complex countries in 2016: Democratic Republic of the Congo, Congo, Chad, Central African Republic, Equatorial Guinea, Liberia, Gabon, Guinea, South Sudan, Nigeria, Burkina Faso, Benin, Mozambique, Angola, Sierra Leone, and Madagascar. In comparison, ‘motorcyclist road injuries’ is an ubiquitous disease-type (81 countries have an RDD in this disease-type in 2016), mostly prevalent in more diversified countries. While ‘appendicitis’ is an ubiquitous disease (it counts 95 countries with RDD), that is more prevalent in non-diversified countries.

From Figs 4 and 5, we can see that high-HCI countries tend to be richer and high-DCI diseases tend to be prevalent in higher-income countries. We develop further this point in the next subsection.

### The HCI by income group of countries and over time

**Fig 8:**
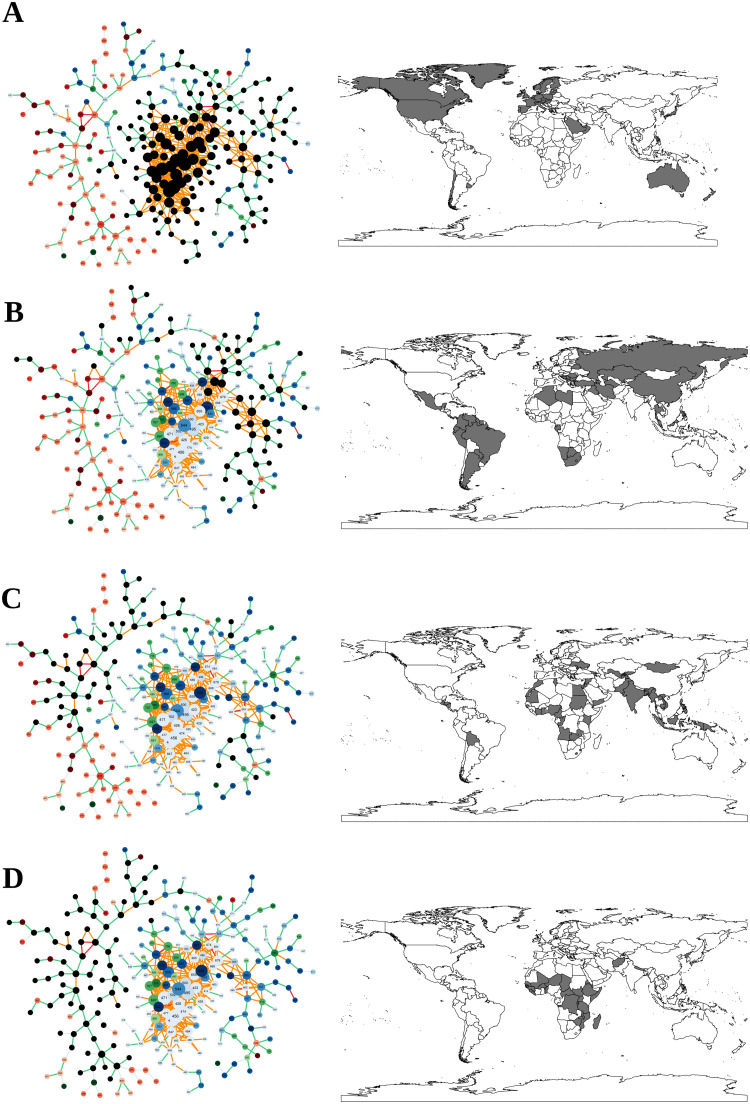
Localisation of diseases for different income-regions of the world. A: High income, B: Upper-middle income, C: Lower-middle income, D: Low income. Diseases in an income-region where more than half of the countries belonging to this region have an RDD are shown with black nodes in the disease space network. Data for 2016.

Fig 8 shows the patterns of disease localization in the world’s economies, classified by the World Bank into four income groups: ‘high’, ‘upper-middle’, ‘lower-middle’, and ‘low’. Diseases in a region where more than half of the countries have an RDD ≥ 1 are shown with black nodes. It seems that high-income countries occupy the core, composed of ‘non-communicable diseases’ such as ‘pancreatic cancer’, ‘Parkinson disease’, ‘ischemic stroke’, and injuries such as ‘falls’, ‘poisonings’ and ‘other exposure to mechanical forces’. On the other side of the spectrum, low-income countries tend to have an RDD in ‘communicable, neonatal, maternal and nutritional diseases’ that lie in the periphery of the disease space, such as ‘diarrheal diseases’, ‘encephalitis’, and ‘malaria’. Most of the communicable diseases for which low-income countries have an RDD ≥ 1 also appear in the periphery (for example, ‘Turner syndrome’, ‘neural tube defects’, and ‘pyoderma’). Examples of injuries for which low-income countries have an RDD include ‘venomous animal contact’ and ‘sexual violence’, which again appear in the periphery of the disease space.

Previous research shows that there is a strong association between income and indicators of population health such as life expectancy and child mortality. There are various channels through which economic development can stimulate health improvements, for example, via its effect on nutrition (which in turn leads to better resistance to bacterial diseases and faster recovery from illnesses), as well as through greater labor market participation, worker productivity, investment in human capital, investment in public and private health services, savings, fertility, transportation infrastructure, lifestyle habits, and innovation in medical treatments [3, 37, 77–85]. For a review of the empirical evidence see [86].

There have also been negative impacts from development, as shown by increases in injuries (e.g., road injuries), heart diseases (e.g., coronary artery disease), and diseases resulting from environmental degradation [87]. Due to modern lifestyles, populations in developed countries are bound to engage in some unhealthy behaviors that lead to health-related problems like obesity, hypertension, diabetes, cancers, and respiratory diseases. These non-communicable diseases, called ‘diseases of affluence’ or ‘diseases of civilization’ correspond to more diversified and less ubiquitous diseases in our analysis. It has been shown that they are dominant in all middle and high income countries and that their main determinant is economic development [17, 20, 37, 88, 89–91].

On the other side, the so-called ‘diseases of poverty’ include mostly communicable diseases that largely result from and contribute to human impoverishment. In our analysis, these diseases are non-complex, i.e., are prevalent infrequently in only few non-diversified countries. Their determinants/risks include some familiar enemies of health and allies of poverty, such as unsafe water, poor sanitation and hygiene, underweight, and indoor smoke from solid fuels [20]. Many people in the developing world, particularly women and children, continue to suffer from undernutrition. According to WHO [20] poverty is the main underlying determinant of undernutrition, and 50-70% of the burden of (non-complex) communicable diseases like diarrheal diseases, malaria, and lower respiratory infections in childhood is attributable to undernutrition. Among adolescents and adults, undernutrition is also associated with adverse pregnancy outcomes, which is one of the five lowest DCI disease sections in our dataset (Table 1).

**Fig 9:**
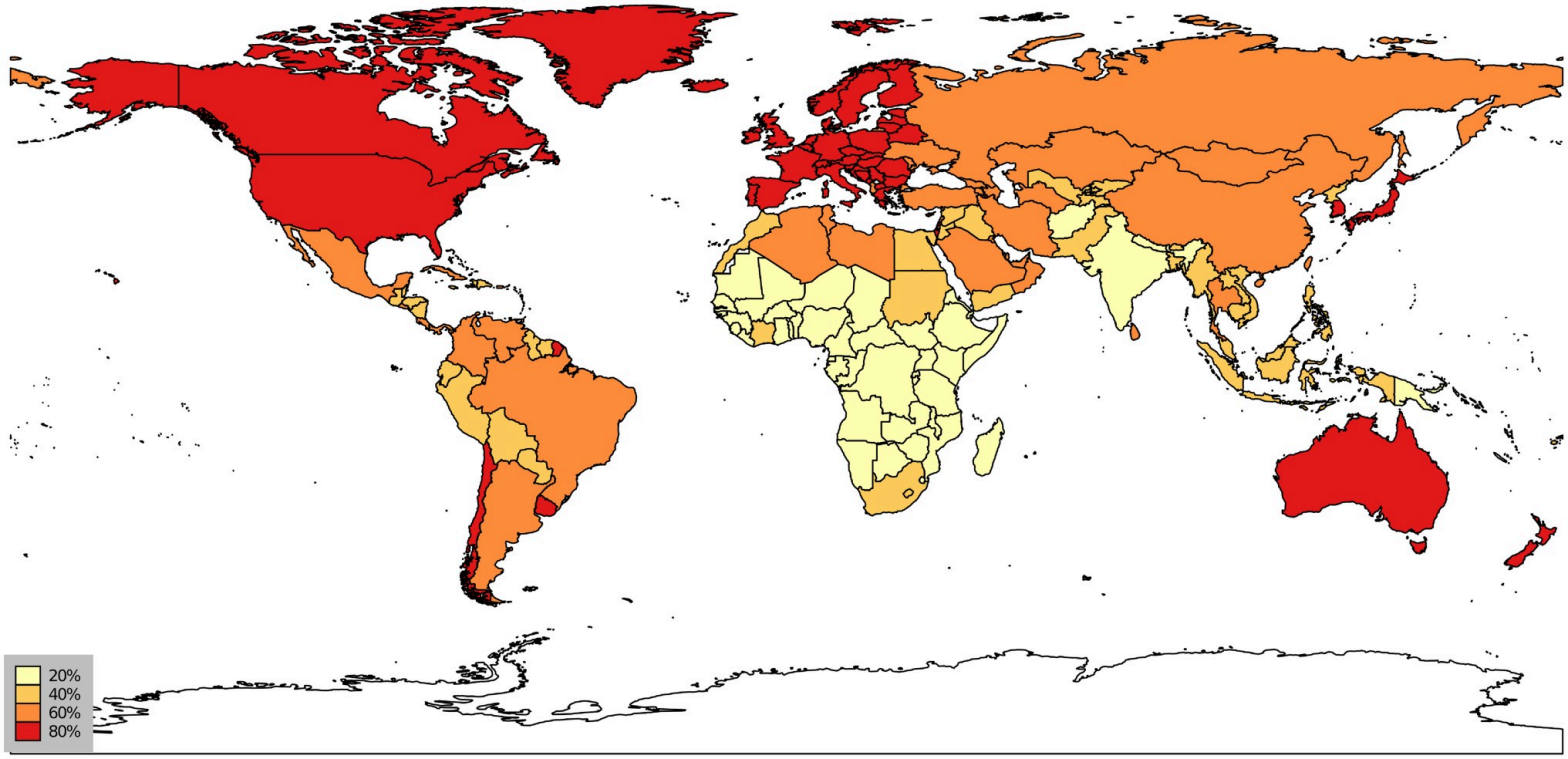
Health complexity index across the world. Percentile repartition. Countries depicted in dark red have an HCI above the 80th percentile. Data for 2016.

The descriptive findings in Fig 8 and the above discussion are also observable in Fig 9, where we map the spatial variation in complex diseases. This figure shows the repartition of the HCI across countries in 2016. We see rather clearly that disease complexity is unevenly distributed in the world and that the most complex countries in terms of diseases seem to be located in Europe, North America, and Australia—European countries, Australia, the US, and Canada belong to the set of countries with the highest HCI (> 80%). In contrast, most countries in Africa have much lower HCIs on average.

**Fig 10:**
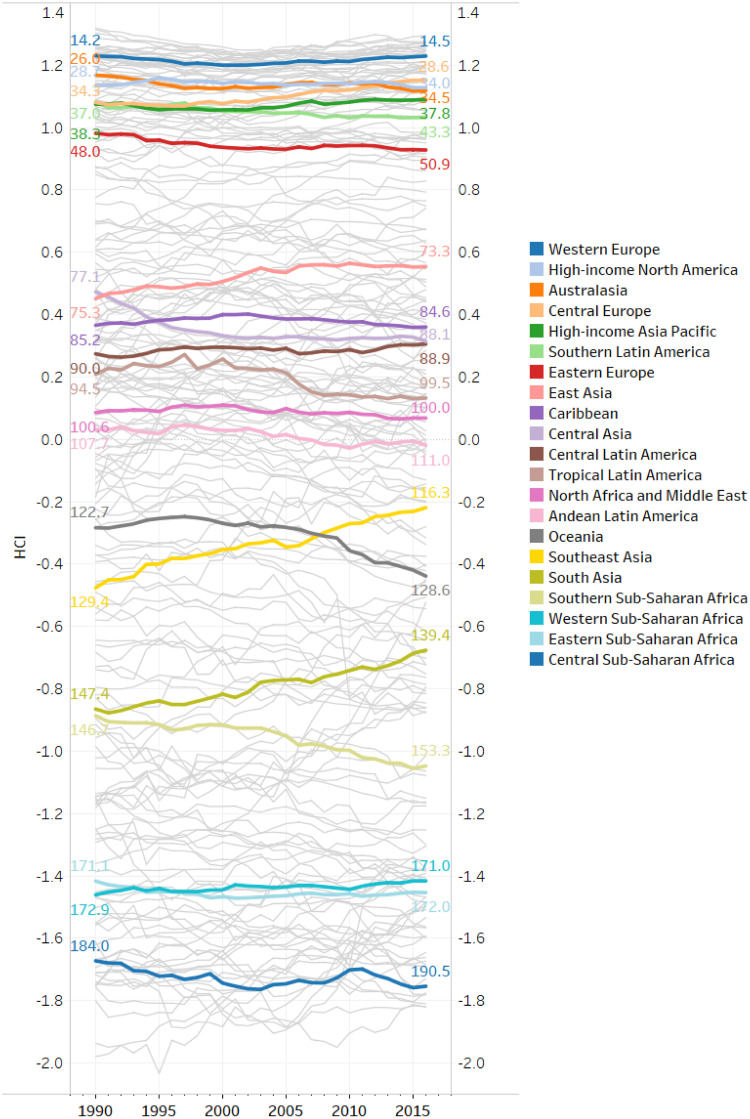
Evolution of the health complexity index (HCI) between 1990 and 2016. The greyed out lines represent the evolution of the HCI for each of the 195 countries. The colors depict different sub-national regions of the world according to the Global Burden of Diseases’ classification. The colored lines display the yearly average of countries’ HCI and the labels the average of countries’ HCI rank in 1990 and 2016 by sub-national region.

Regarding the evolution of HCI scores over time, Fig 10 depict the HCI scores by country and for different sub-national regions of the world from 1990 to 2016. As observed in Fig 9 for 2016, the countries in Western Europe, North America, and Australasia have the highest HCIs in the world and these scores remain stable over the period covered. Countries located in High-income Asia Pacific, Southern Latin America, and Eastern Europe followed a similar pattern. Amongst the high-HCI regions, Central Europe had the highest increase in the complexity of its diseases. It gained 5 positions on average in the HCI ranking (from 34.3 in 1990 to 28.6 in 2016), with Poland climbing up to 20 positions between 1990 and 2016. In regions with upper-middle HCIs in 2016, we observe a slight increase in the complexity of diseases, in particular for countries in East Asia, Caribbean, Central Latin America, and North Africa and Middle East. They gained 1 to 3 positions on average in the HCI ranking between 1990 and 2016. On the other hand, the diseases of countries in Andean and Tropical Latin America, and Central Asia are now less complex than in 1990. Central Asia for example, lost 11 positions, and Tropical Latin America 5 positions, between 1990 and 2016.

In contrast, the variation in HCI is stronger for countries with a lower-middle HCI in 2016. Southeast and South Asia experienced a significant increase in the complexity of their diseases. Countries in Southeast Asia ranked 129.4 on average in 1990 compared to 116.3 in 2016. Myanmar, Malaysia, Mauritius Myanmar, Sri Lanka, Thailand, and Vietnam gained from 10 to 20 positions between 1990 and 2016. While countries in South Asia, such as Bangladesh and Nepal, gained 8 positions on average in the period covered. On the other hand, the HCI decreased in Oceania, mainly because of a strong decrease in Vanuatu (that lost 20 positions in the HCI ranking), Kiribati (lost 10 positions) and Papua New Guinea (lost 8 positions), and in Southern Sub-Saharan Africa, in particular South Africa. For countries with low HCI in 2016, the index remains rather stable over time. We note a slight increase in health complexity for Western Sub-Saharan Africa. Cameroon, for instance, gained 19 positions in the HCI ranking between 1990 and 2016. In Central Sub-Saharan Africa, where the HCI scores are among the lowests, we observe a decrease in health complexity over time. Indeed, countries such as Equatorial Guinea lost 11 positions in the HCI ranking, and Gabon 20 positions between 1990 and 2016.

In total, Fig 10 shows that changes over time are rather small. Hence, it seems that a country’s HCI tends to persist through time, which is to be expected for a metric of disease prevalence aggregated at the country level. This motivates the inclusion of the lagged value of the HCI in the set of explanatory variables when exploring the link between economic development and health complexity in the next section.

## The effect of economic development on health complexity

We study the effect of economic development on health complexity using data on GDP per capita (from the World Bank’s World Development Indicators) and the HCI (see Section ‘Methods’). Given the availability of controls, the sample covers a minimum of 168 developed and developing countries over the period of 1992-2015. For more information, see S1 Appendix: List of countries in regressions.

### Regression analysis

In order to estimate the effect of economic development on the health complexity of countries, we follow a fixed-effects two-stage least squares/instrumental variables (FE 2SLS/IV) strategy, complemented with a difference Generalized Method of Moments (diff-GMM) approach. The regression analysis was performed with Stata/SE 16.1. We regress the baseline specification described by the following equation:

$$\begin{array}{c} {HCI}_{i,t}=\rho {HCI}_{i,t-1}+\beta_{1}GDPpc_{i,t}+\beta_{k}controls_{i,t}+\gamma_{i}+\delta_{t}+u_{i,t}. \end{array}$$ (7)

Here, the health complexity of country *i* in period *t* (*HCI*_*i*,*t*_) depends on the country’s level of economic development in per capita terms (in logs), *GDPpc*_*i*,*t*_. The lagged value of the dependent variable on the right-hand side is included to capture persistence in health complexity. The main variable of interest is *GDPpc*. The parameter *β*_1_ therefore measures the link between income per capita and health complexity. As discussed above, the set of diseases in a country does not increase monotonically in time. When countries become more developed, they are more effective at fighting diseases therefore some diseases disappear. In order to account for possible changes in the relation between income per capita and health complexity over the process of economic development (turning point), we have experimented with the inclusion of the quadratic specification of GDP per capita in the estimated equation. Our baseline results (which are available upon request) do not confirm an U-shaped relationship between economic development and health complexity. Additional potential covariates are included in the vector *controls*_*i*,*t*_. The *γ*_*i*_’s denote a full set of country dummies and the *δ*_*t*_’s denote a full set of time effects that capture common shocks to the health complexity scores of all countries. The error term *u*_*i*,*t*_ captures all other omitted factors, with *E*(*u*_*i*,*t*_) = 0 for all *i* and *t*.

To examine the robustness of our results and to generalize our findings, we replicate our analysis for additional/alternative control variables and substitute the HCI with the AHCI, finding qualitatively similar results (see subsection).

#### Control variables

We include in the estimated equation a number of control variables that are likely related to health complexity.

The proportion of nations’ populations over the age of 65 has been increasing in recent years and will continue to rise in future as a result of longer life expectancy. Population age-structure is a significant determinant of a nation’s health status, due to age-related diseases (i.e., illnesses and conditions that occur more frequently in people as they get older). Examples of age-related diseases include cardiovascular and cerebrovascular diseases, hypertension, cancer, Parkinson’s disease, Alzheimer’s disease, osteoarthritis and osteoporosis. Demographic factors such as age and sex are considered key covariates in the study of human health and well-being, hence the percentage of *old* population (aged 65 and above, in logs) and the percentage of *female* population (in logs) are included in the set of control variables.

It has previously been shown that sex interacts with social, economic and biological determinants to create different health outcomes for males and females. For example, [92] reviews a large number of studies on the interaction between sex and the determinants and consequences of chronic diseases, showing how these interactions result in different approaches to prevention, treatment, and coping with illness.

In our analysis, we also control for the (log) percentage of urban population, *urban*. According to the World Health Organization (WHO), a large proportion of non-communicable diseases is linked to risks related to the urban environment, such as physical inactivity and obesity, cardiovascular and pulmonary diseases from transport-generated urban air pollution, ischemic heart disease and cancers from household biomass energy use, asthma from indoor air pollution, and heat-related strokes and illnesses. In addition, communicable diseases such as tuberculosis, dengue fever, and many respiratory and diarrheal diseases result from unhealthy urban environments (e.g. lack of adequate ventilation, unsafe water storage and poor waste management, indoor air pollution, moldy housing interiors, poor sanitation). See [93].

Industrialization, i.e., the structural transformation from agricultural to industrial production also has a range of significant health implications [94–98]. We capture these implications in our analysis by including the (log) value added of *agriculture* (% of GDP) and the (log) value added of *manufacturing* (% of GDP) in the estimated equation.

To check the robustness of our baseline results, we replicate our analysis controlling also for the human capital of the population by using total enrolment in tertiary *education* (in logs). It is well established in the relevant literature that through education, people gain the ability to be effective in their lives. They adopt healthier lifestyles and inspire their offspring to do as well [99]. Individuals with higher levels of education also tend to have better socioeconomic resources for a healthy lifestyle and a higher probability of living and working in healthy environments [100, 101]. In addition, educated individuals tend to have lower exposure to chronic stress [102]. Low educational attainment, on the other hand, is associated with a shorter life expectancy, poor self-reported health, and a high prevalence of infectious and chronic non-infectious diseases [103–106]. [107] and [108] review the relationships between education and a wide variety of health measures. Furthermore, we re-estimate the baseline model by substituting *urban* with *population* *density* (people per square km of land, in logs).

The variable *CO*_2_ (*CO*_2_ emissions in kg per 2010 $US of GDP) captures the effect of air pollution on health, which has been the subject of numerous studies in recent years (for an extensive review, see [109]).

Finally, *health* *expenditure* (log of total health spending in thousands of purchasing power parity (PPP)-adjusted 2017 $US) is also included in the set of explanatory variables controlling for the association between healthcare spending and health outcomes [110–117]

Data definitions, sources and summary statistics for the variables included in the analysis are given in Table 2.

**Table 2:** Variable definitions, sources and summary statistics.

Variable	Definition	Source	Mean	Std. Dev.
HCI	Health Complexity Index.	Authors’ calculations	-0.006	1.004
HCI+	age-standardized Health Complexity Index.	Authors’ calculations	-0.008	1.008
GDPpc	(log) GDP per capita, PPP (constant 2011 international $)	World Development Indicators	8.342	1.521
old	(log) Population aged 65 and above (% of total)	World Development Indicators	1.764	0.667
female	(log) Female population (% of total)	World Development Indicators	3.912	0.067
urban	(log) Urban population (% of total)	World Development Indicators	3.871	0.519
agriculture	(log) Agriculture, value added (% of GDP)	World Development Indicators	2.054	1.228
manufacturing	(log) Manufacturing, value added (% of GDP)	World Development Indicators	2.407	0.591
education	(log) Enrollment in secondary education, both sexes (total)	World Development Indicators	4.229	0.587
population density	(log) Population density (people per sq. km of land area)	World Development Indicators	4.123	1.398
CO_2_	(log) CO_2_ emissions (kg per 2010 $US of GDP)	World Bank	-0.930	0.704
health expenditure	(log) Total health spending, PPP (thousands of 2017 $US)	Global Health Spending 1995-2015, IHME	15.071	2.239
economic globalization	Actual flows (trade, foreign direct investment, stocks, portfolio investment, income payments to foreign nationals), restrictions (hidden import barriers, mean tariff rate, taxes on international trade, capital account restrictions). Higher values reflect greater economic globalization.	KOF Index of Globalization	54.462	16.234
political globalization	Embassies in country, membership in international organizations, participation in UN security council missions, international treaties. Higher values reflect greater political globalization.	KOF Index of Globalization	62.283	21.985

#### Instrumental variables

We estimate Eq (7) using different econometric methods. First, we use fixed-effects OLS. However, fixed effects estimators do not necessarily identify the effect of economic development on health complexity. The estimation of causal effects requires exogenous sources of variation. While we do not have an ideal source of exogenous variation recognized by previous studies, there are two promising potential instruments of economic development that we adopt in our fixed-effects 2SLS/IV and diff-GMM analyses.

First, we use the KOF Swiss Economic Institute’s *economic* *globalization* index, characterized as the flows of goods, capital, and services, as well as information and perceptions that accompany market exchanges [118]. Higher values reflect greater economic globalization.

The second instrument considered is the KOF Swiss Economic Institute’s *political* *globalization* index, characterized by the number of embassies in a country, its membership in international organizations, its participation in UN security council missions and international treaties. Higher values reflect greater political globalization.

There is extensive research documenting the positive relationship between globalization and economic development and growth [118–122]. While we do not develop a theoretical model of supporting the HCI in this paper, we expect that changes in the *economic* *globalization* and *political* *globalization* indices have no direct effect on a country’s health complexity. We predict that both indices of globalization impact the HCI only indirectly, through the channel of economic development. This prediction is verified in our dataset by a series of statistical tests (see next section).

### Regression results

In this section, we discuss the results of estimating Eq (7) with different econometric techniques. Table 3 reports the results of fixed-effects ordinary least squares (FE-OLS) with time dummies, adding an additional variable from the set of controls in each step (column). In all specifications, economic development has a positive relationship with health complexity, and the control variables enter with the expected sign. The *agriculture* coefficient is negative, and countries with a higher proportion of *urban* population exhibit greater health complexity.

**Table 3:** The effect of economic development on health complexity: Fixed-effects OLS.

	(1)	(2)	(3)	(4)	(5)	(6)
*HCI*_*t*−1_	0.938***	0.938***	0.938***	0.933***	0.929***	0.929***
	(0.009)	(0.009)	(0.010)	(0.010)	(0.010)	(0.010)
*GDPpc*	0.013***	0.013***	0.013***	0.012***	0.009**	0.011***
	(0.003)	(0.003)	(0.003)	(0.003)	(0.003)	(0.004)
*old*		-0.001	-0.001	0.002	0.000	0.002
		(0.006)	(0.006)	(0.006)	(0.007)	(0.007)
*female*			-0.014	-0.013	0.002	-0.006
			(0.024)	(0.024)	(0.023)	(0.024)
*urban*				0.020**	0.025***	0.021**
				(0.008)	(0.008)	(0.009)
*agriculture*					-0.006***	-0.006***
					(0.002)	(0.002)
*manufacturing*						0.001
						(0.002)
Observations	4,750	4,593	4,593	4,593	4,377	4,181
Countries	190	183	183	183	180	177
R-squared	0.873	0.874	0.874	0.875	0.872	0.872

Note: Dependent variable: Health Complexity Index (HCI). Main independent variable: GDP per capita in logs (*GDPpc*). Time fixed-effects are included in all regressions. Robust standard errors are in parentheses.

* p<0.10,

** p<0.05,

*** p<0.01.

The results depicted in Table 3 could only be interpreted as correlations. For a robust analysis accounting for the potential endogeneity problem in the relationship under consideration (discussed above), we use fixed-effects 2SLS/IV estimation techniques complemented with diff-GMM estimations à la Arellano-Bond [123]. Table 4 presents our baseline results.

**Table 4:** The effect of economic development on health complexity: Baseline results.

	(1)	(2)	(3)	(4)	(5)	(6)	(7)	(8)	(9)
	FE 2SLS/IV	FE 2SLS/IV	FE 2SLS/IV	FE 2SLS/IV	FE 2SLS/IV	FE 2SLS/IV	FE 2SLS/IV	FE 2SLS/IV	diff-GMM
*HCI*_*t*−1_	0.926***	0.927***	0.927***	0.923***	0.923***	0.922***	0.922***	0.921***	0.814***
	(0.010)	(0.010)	(0.010)	(0.010)	(0.010)	(0.010)	(0.011)	(0.010)	(0.061)
*GDPpc*	0.031***	0.031***	0.031***	0.031***	0.026**	0.029***	0.030*	0.027**	0.032**
	(0.011)	(0.011)	(0.011)	(0.011)	(0.011)	(0.011)	(0.017)	(0.013)	(0.013)
*old*		-0.003	-0.002	-0.001	-0.003	-0.001	-0.002	-0.001	-0.005
		(0.005)	(0.005)	(0.005)	(0.006)	(0.006)	(0.006)	(0.006)	(0.017)
*female*			-0.015	-0.015	-0.019	-0.029	-0.031	-0.026	0.029
			(0.023)	(0.023)	(0.027)	(0.028)	(0.033)	(0.029)	(0.044)
*urban*				0.013	0.020***	0.017**	0.017**	0.017**	0.031*
				(0.008)	(0.008)	(0.008)	(0.009)	(0.008)	(0.018)
*agriculture*					-0.003	-0.003	-0.002	-0.003	-0.002
					(0.003)	(0.003)	(0.004)	(0.003)	(0.008)
*manufacturing*						0.002	0.002	0.002	-0.003
						(0.002)	(0.002)	(0.002)	(0.012)
Fist-stage results									
*economic* *globalization*	0.003***	0.003***	0.003***	0.003***	0.004***	0.003***	0.005***		
	(0.001)	(0.001)	(0.001)	(0.001)	(0.000)	(0.000)	(0.000)		
*political* *globalization*	0.004***	0.004***	0.004***	0.004***	0.004***	0.005***		0.006***	
	(0.001)	(0.001)	(0.001)	(0.001)	(0.000)	(0.000)		(0.000)	
Observations	4,168	4,143	4,143	4,143	3,976	3,826	3,826	3,926	3,652
Countries	171	170	170	170	170	168	168	173	168
F-test	37.24	37.05	36.31	39.93	56.26	61.41	79.18	76.56	
DWH-test	2.978	2.911	2.785	3.073	2.500	2.646	1.304	1.523	
Weak-id	59.73	54.25	54.88	56.82	97.43	107.0	104.3	152.6	
LM-weakid	71.12	70.95	69.68	75.01	109.7	116.3	72.90	73.98	
Hansen (p-value)	0.590	0.699	0.676	0.897	1.000	0.904			
AR(1)									0.000
AR(2)									0.475

Note: Dependent variable: Health Complexity Index (HCI). Main independent variable: GDP per capita in logs (*GDPpc*). Columns (1)-(8): Fixed-effects 2SLS/IV; HCI is instrumented: To save space, we only include the first-stage estimated coefficients of the instruments. The results for the rest of the variables are available upon request. Column (9): One-step diff-GMM. All regressions include time dummies. Robust standard errors are in parentheses. F-test gives the F-statistic for the joint significance of the instruments in the first stage. DWH-test is the Durbin-Wu-Hausman test of endogeneity of the regressors. Weak-id gives the Cragg-Donald F-statistic for weak identification. LM-weakid gives the Kleibergen-Paap Wald test of weak identification. Hansen (p-value) gives the p-value of the Hansen test of overidentification. AR(1) and AR(2) are the p-values for first- and second-order autocorrelated disturbances.

* p<0.10,

** p<0.05,

*** p<0.01.

In columns (1)-(8), we estimate Eq (7) with FE 2SLS/IV regressions. We use time dummies and robust standard errors (in parentheses). In all cases, *GDPpc* is a positive and statistically significant predictor of health complexity. In fact, we find that an increase in *GDP* *per* *capita* of 10% is associated with an improvement of about 0.003 in the HCI (standard deviation: 1.004). This positive impact of economic development on health complexity is robust to the inclusion of control measures discussed in subsection 1. The statistically significant *urban* coefficient implies that a higher proportion of urban population is associated with more complex diseases.

In the fixed effects 2SLS/IV estimations we report: (a) the *F*-*test* for the joint significance of the instruments in the first stage: the rule of thumb is to exceed 10, hence the test implies weak significance [124]; (b) the Durbin-Wu-Hausmann (*DWH*) test for the endogeneity of regressors: the null hypothesis that the IV regression is not required is rejected; (c) the Cragg-Donald F-statistic (*Weak*-*id*), testing the relevance of the instruments in the first-stage regression: no evidence of a low correlation between instruments and the endogenous regressor is found after controlling for the exogenous regressors; (d) the Kleibergen-Paap Wald test (*LM*-*weakid*) of weak identification: the null hypothesis that the model is weakly identified is rejected; (e) the p-value for Hansen’s test of overidentification: the acceptance of the null indicates that the overidentifying restrictions cannot be rejected.

In column (9) of Table 4 we report the diff-GMM estimations including year fixed effects and robust standard errors. The results verify the previous findings both qualitatively and quantitatively, i.e., the estimated coefficient of *GDPpc* implies an increase of 0.003 in the HCI with a *GDP* *per* *capita* increase of 10%. Among the control variables, only the *urban* variable has a statistically significant and positive sign. The values reported for AR(1) and AR(2) are the p-values for first- and second-order autocorrelated disturbances. As expected, there is high first-order autocorrelation and no evidence for significant second-order autocorrelation. Hence, our test statistics hint at a proper specification.

In Tables 5 and 6, we investigate the robustness of our baseline findings. First, we substitute the HCI with the age-standardized health complexity index (AHCI) maintaining the same set of controls (and time dummies) as in the baseline specification. Second, we investigate whether the positive impact of economic development on health complexity persists under additional and/or alternative control measures (including time dummies). In all cases, the baseline results remain qualitatively intact. In particular, the coefficient of *GDPpc* is positive and statistically significant in the instrumented regressions (see Table 5; to save space, we only include the first-stage estimated coefficients of the instruments—the results for the rest of the variables are available upon request).

**Table 5:** The effect of economic development on health complexity: AHCI.

	(1)	(2)	(3)	(4)
	FE 2SLS/IV	FE 2SLS/IV	FE 2SLS/IV	diff-GMM
*AHCI*_*t*−1_	0.923***	0.925***	0.919***	0.828***
	(0.011)	(0.012)	(0.011)	(0.114)
*GDPpc*	0.037***	0.033**	0.041***	0.024*
	(0.011)	(0.015)	(0.014)	(0.013)
*female*	-0.028	-0.021	-0.037	0.030
	(0.027)	(0.034)	(0.031)	(0.040)
*urban*	0.009	0.010	0.007	0.011
	(0.007)	(0.008)	(0.008)	(0.023)
*agriculture*	0.002	0.001	0.003	0.008
	(0.003)	(0.004)	(0.004)	(0.010)
*manufacturing*	0.002	0.002	0.003	-0.013
	(0.002)	(0.002)	(0.002)	(0.011)
Fist-stage results				
*economic* *globalization*	0.004***	0.005***		
	(0.000)	(0.000)		
*political* *globalization*	0.004***		0.005***	
	(0.000)		(0.000)	
Observations	3,826	3,826	3926	3,652
Countries	168	168	173	168
F-test	67.39	101.5	71.22	
DWH-test	6.055	2.200	4.548	
Weak-id	116.2	136.4	138.9	
LM-weakid	125.8	92.43	67.20	
Hansen (p-value)	0.681			
AR(1)				0.000
AR(2)				0.158

Note: Dependent variable: Age-standardized Health Complexity Index (AHCI). Main independent variable: GDP per capita in logs (*GDPpc*). Columns (1)-(3): Fixed-effects 2SLS/IV; *AHCI* is instrumented: To save space, we only include the first-stage estimated coefficients of the instruments. The results for the rest of the variables are available upon request. Column (4): One-step diff-GMM. All regressions include time dummies. Robust standard errors are in parentheses. F-test gives the F-statistic for the joint significance of the instruments in the first stage. DWH-test is the Durbin-Wu-Hausman test of endogeneity of the regressors. Weak-id gives the Cragg-Donald F-statistic for weak identification. LM-weakid gives the Kleibergen-Paap Wald test of weak identification. Hansen (p-value) gives the p-value of the Hansen test of overidentification. AR(1) and AR(2) are the p-values for first- and second-order autocorrelated disturbances.

* p<0.10,

** p<0.05,

*** p<0.01.

**Table 6:** The effect of economic development on health complexity: Robustness checks.

	(1)	(2)	(3)	(4)	(5)
	diff-GMM	diff-GMM	diff-GMM	diff-GMM	diff-GMM
*HCI*_*t*−1_	0.752***	0.871***	0.812***	0.920***	0.666***
	(0.059)	(0.046)	(0.063)	(0.040)	(0.062)
*GDPpc*	0.031*	0.031***	0.024*	0.042***	0.038*
	(0.016)	(0.011)	(0.013)	(0.016)	(0.020)
*old*	-0.022	-0.010	0.004	-0.002	-0.005
	(0.024)	(0.017)	(0.018)	(0.016)	(0.029)
*female*	0.053	0.018	0.053	-0.068	-0.170
	(0.050)	(0.051)	(0.045)	(0.047)	(0.124)
*urban*	0.055		0.013	0.022	
	(0.035)		(0.017)	(0.023)	
*agriculture*	0.007	-0.001	0.003	0.001	0.015
	(0.009)	(0.006)	(0.009)	(0.008)	(0.011)
*manufacturing*	0.008	-0.003	-0.015	0.014	0.020
	(0.012)	(0.011)	(0.010)	(0.011)	(0.014)
*education*	-0.003				0.026
	(0.011)				(0.017)
*population* *density*		0.000			-0.073
		(0.016)			(0.049)
*CO*_2_			0.013*		-0.003
			(0.007)		(0.015)
*health* *expenditure*				-0.006	-0.012
				(0.012)	(0.018)
Observations	2,347	3,642	3,470	3,158	1,936
Countries	161	168	168	168	158
AR(1)	0.000	0.000	0.000	0.000	0.000
AR(2)	0.395	0.505	0.621	0.525	0.274

Note: Dependent variable: Health Complexity Index (HCI). Main independent variable: GDP per capita in logs (*GDPpc*). Regression analysis: One-step diff-GMM. All regressions include time dummies. Robust standard errors are in parentheses. AR(1) and AR(2) are the p-values for first- and second-order autocorrelated disturbances.

* p<0.10,

** p<0.05,

*** p<0.01.

Table 6 starts from the baseline specification with the full set of controls [column (9) in Table 4] and introduces additional variables or alternative measures for some of the previous controls. Specifically, in column (1), we add *education* (enrolment in secondary education in logs). In column (2), we substitute the *urban* population variable with *population* *density* (people per sq. km of land in logs). In columns (3) and (4), we employ (log) *CO*_2_ emissions (*CO*_2_ emissions, kg per 2010 $US of GDP) and (log) *health* *expenditure* (total health spending, thousands of 2017 PPP adjusted $US), respectively. Finally, in column (5), we consider all of the above variables together. Adding these controls in our estimations leaves the findings qualitatively and quantitatively intact.

The above analysis suggests that economically developed countries tend to exhibit more complex disease structures. Furthermore, exploiting the temporal variation in the data, the fixed-effects 2SLS/IV analysis and the difference GMM estimators reveal a positive, statistically significant, and robust impact of economic development on health complexity.

## Economic development and disease complexity

The economic complexity methodology provides a useful toolbox that allows us to compute indices that quantify the complexity of both countries and diseases. The DCI quantifies the complexity of countries’ diseases according to their rate of prevalence worldwide.

Using the economic complexity methodology, [56] recently introduced a measure that associates products with income inequality and showed how the development of new products is associated with changes in income inequality. Here, to decompose economic development at the disease level, we introduce a measure that links a disease to the average income per capita of the countries in which the disease is prevalent i.e., an estimate of the expected income per capita related to different diseases. In this way, we illustrate how disease complexity is being affected by the level of economic development and quantify the relationship between countries’ income per capita and the complexity of their diseases.

Following the methodology in [56], we define the *Disease-Income Complexity Index* (DICI) and decompose the relationship between the DCI and the DICI for the diseases in our sample of countries. We also computed the Age-standardized Disease-Income Complexity Index ADICI and investigated its relationship with the ADCI, finding similar results.

### The disease-income complexity index

Assuming that we have information for *l* countries and *k* diseases, we can fill the (*l* × *k*) matrix **M** so that its matrix element *M*_*cd*_ = 1 if country *c* has an RDD in disease *d*, and zero otherwise (see Section ‘The relative disease disadvantage’). Our dataset contains information for 195 developed and developing countries and for 196 diseases from 1990 to 2016.

Every disease *d* can have prevalent cases in a country *c*. For every disease *d*, we can calculate the fraction *s*_*cd*_:

$$\begin{array}{c} s_{cd}=\frac{X_{cd}}{\sum_{d^{\prime }}X_{cd^{\prime }}}, \end{array}$$ (8)

where *X*_*cd*_ is the number of cases per 100,000 population for disease *d* in country *c*, while ∑_*d*′_ *X*_*cd*′_ is the number of cases of all diseases in country *c*. If *GDP*_*c*_ is the (log) *GDP* *per* *capita* of country *c*, we can calculate the DICI_*d*_ for every disease *d* as:

$$\begin{array}{c} {\text{DICI}}_{d}=\frac{1}{N_{d}}\sum_{c}M_{cd}s_{cd}GDP_{c}, \end{array}$$ (9)

where *N*_*d*_ = ∑_*c*_ *M*_*cd*_ *s*_*cd*_ is a normalization factor.

The DICI is defined at the disease level as the average level of (log) *GDP* *per* *capita* of the countries that have an RDD in disease *d*, weighted by the disease’s importance in each country’s pool of diseases. Using the (log) PPP *GDP* *per* *capita* (constant 2011 international $) from the World Bank’s World Development Indicators for the countries in our sample, we calculate the above index for every year in the period of 1990-2016.

Table 7 lists the five diseases with the highest and lowest average DICI values during the period of 1990-2016. It is evident that higher economic development is associated with more complex diseases such as motor neuron disease and malignant skin melanoma. At the other end of the spectrum, less complex diseases such as acute hepatitis E and malaria are associated with low levels of income per capita.

**Table 7:** List of the five diseases with the highest and lowest DICI values during the period of 1990-2016.

code	disease name	disease section	DICI	DCI
*Highest DICI*				
554	Motor neuron disease	Neurological disorders	4.55	1.025
459	Malignant skin melanoma	Neoplasms	4.52	1.210
573	Anorexia nervosa	Mental disorders	4.49	0.818
483	Mesothelioma	Neoplasms	4.49	1.041
485	Non-Hodgkin’s lymphoma	Neoplasms	4.48	1.057
*Lowest DICI*				
404	Acute hepatitis E	Other infectious diseases	2.89	-1.707
345	Malaria	Neglected tropical diseases and malaria	2.86	-2.045
353	Cystic echinococcosis	Neglected tropical diseases and malaria	2.84	-1.383
370	Maternal obstructed labor and uterine rupture	Maternal and neonatal disorders	2.80	-1.957
359	Rabies	Neglected tropical diseases and malaria	2.76	-1.840

Notes: DICI: Disease-Income Complexity Index; DCI: Disease Complexity Index; Average values for 1990-2016

### Linking economic development and disease complexity

In this subsection, we test the existence of a bivariate relationship between the DCI and the DICI. Thus, we calculate Pearson’s correlation coefficient for DICI against DCI. If such an association exists, it should allow us to derive expectations about whether disease complexity can be associated with economic development and verify, with disease-level data, the statistically significant and positive relationship between health complexity and economic development that we found above (Section 1). The correlation coefficient for the relationship between the average values of the DICI and the DCI for the period of 1990-2016 is *ρ* = 0.96 ± 0.01 with a p-value <2.2 × 10^−16^.

**Fig 11:**
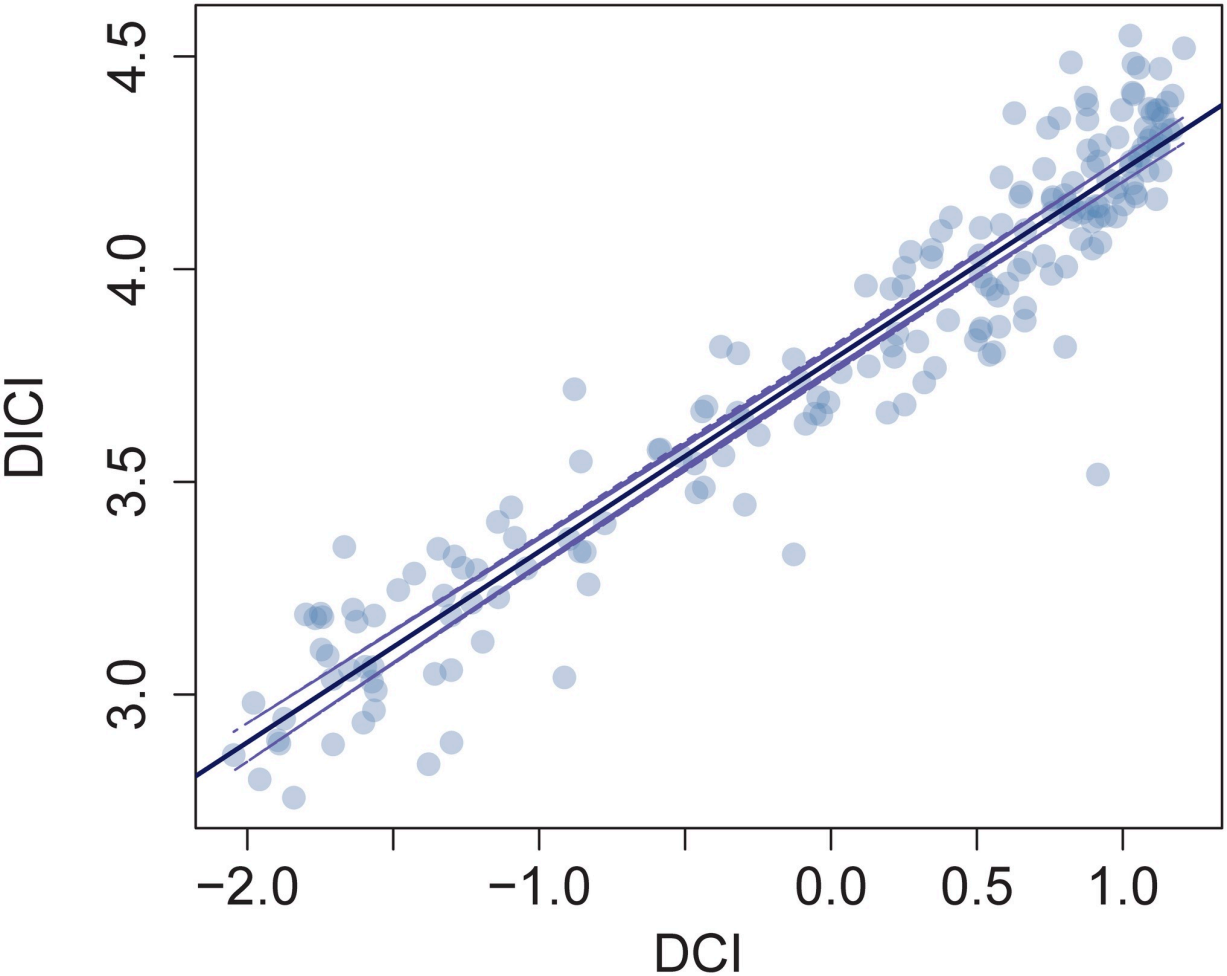
DICI against DCI. The solid line represents the fit of a linear model and the dashed line a 95% prediction interval based on the fitted linear model.

In Fig 11, we present the scatter-plot of the relationship between the DICI and the DCI for the 196 diseases in our dataset (average values for 1990-2016), together with the fitted linear model. The slope of the linear fit is the corresponding correlation coefficient.

The statistically significant positive correlation between the DICI and the DCI indicates that more complex diseases are associated with more developed countries, as measured by the (log) *GDP* *per* *capita*. This allows us to understand which sets of diseases are linked to better overall economic performance, based on their complexity.

In Table 8, we run panel regressions between the DCI and DICI. The results show that the relationship between the DCI and the DICI is the outcome of the correlations both *between* diseases (regression on group means) and *within* diseases (fixed-effects regression with time dummies and standard errors adjusted for disease clusters). Running panel regressions in this way allows us to exploit both the time-series and cross-sectional information in the data and in our case suggests that the positive association of economic development with the complexity of diseases is due to changes in the structure of the disease space towards more complex diseases across time (the disease complexity increases over time) and between diseases (the disease complexity increases in relative terms).

**Table 8:** Disease-income complexity index and the complexity of diseases.

	(1)	(2)
	DCI	DCI
	Within Estimation	Between Estimation
DICI	0.479***	2.041***
	(0.149)	(0.045)
Observations	5,211	5,211
Diseases	193	193
R-square	0.90	0.88

Notes: DICI: Disease-Income Complexity Index; DCI: Disease Complexity Index. Country and time dummies are included in the within regression. Standard errors are in parentheses.

* *p* < 0.10,

** *p* < 0.05,

*** *p* < 0.01

## Conclusions

Our analysis illustrates that the development of nations conditions the structure of the disease space. Following the economic complexity methodology, we developed the HCI (DCI), which quantifies the network representation of the co-occurrence of diseases. In a dynamic panel data setting, we showed that there is a positive relationship between a country’s economic development, measured by the GDP per capita, and its level of health complexity, i.e., the ‘structural’ composition of its pool of diseases. Specifically, more complex diseases tend to be prevalent in populations with a higher income per capita.

Explicitly, the HCI and DCI reveal the following pattern across countries: High-DCI diseases, i.e., the non-ubiquitous diseases that tend to be prevalent in the more disease-diversified (high-HCI) countries, tend to be non-communicable diseases. Moreover, high-HCI countries, which are the countries with RDD in high-DCI diseases, tend to be richer. Low-DCI diseases, i.e., the non-ubiquitous diseases that tend to be prevalent in the less-diversified (low-HCI) countries, tend to be communicable diseases. Moreover, low-HCI countries, which are the countries with RDD in low-DCI diseases, tend to be poorer.

In addition, we built the DICI, which links a disease to the average level of income per capita of the countries in which the disease is prevalent, and illustrate how disease complexity is related to economic development. Specifically, we showed how changes in GDP per capita are associated with more high-DCI diseases. The temporal variation of the above indices is important from a policy perspective. Using the HCI and DCI, it is possible to design policies aimed at improving the recognition, visibility, and traceability of high-DCI diseases across the globe and through time (e.g., by developing a classification system for all health information systems). These indices can also be used as tools for the development of national plans and the establishment of knowledge networks for complex diseases. Furthermore, the DICI could be used to design a health expenditure reallocation policy promoting health activities and services associated with the prevention of complex diseases.

In sum, this study employs the economic complexity methodology to compute two new metrics that quantify the disease space of countries. These unified measures can be valuable tools to monitor, study the determinants, and measuring the impact of public policies on the evolution of countries’ structure of diseases and the distribution of diseases worldwide. The topic of economic complexity is a rather new one, and its use in health economics is so far rather limited. By focusing on the topic of disease complexity, our contribution lies in bridging the health economics literature with the literature that highlights economic complexity as a powerful paradigm in understanding key issues in economics, geography, innovation studies, and other social sciences.

## Supporting information

S1 Table List of countries in the dataset. (PDF)

S2 Table List of diseases and injuries in the dataset. (PDF)

S1 Appendix List of countries in regressions. (PDF)
