# Supplementary material for: The development of nations conditions the disease space
Source: PLoS One. 2021 Jan 7;16(1):e0244843. doi: 10.1371/journal.pone.0244843 (PMC7790431; doi:10.1371/journal.pone.0244843)
Supplement: S1 Table — (PDF) [file pone.0244843.s001.pdf]

S1 Table. List of countries in the dataset.

Country id	Country name	Region	World Bank group
6	China	East Asia	World Bank Upper Middle Income
7	North Korea	East Asia	World Bank Low Income
8	Taiwan	East Asia	World Bank High Income
10	Cambodia	Southeast Asia	World Bank Lower Middle Income
11	Indonesia	Southeast Asia	World Bank Lower Middle Income
12	Laos	Southeast Asia	World Bank Lower Middle Income
13	Malaysia	Southeast Asia	World Bank Upper Middle Income
14	Maldives	Southeast Asia	World Bank Upper Middle Income
15	Myanmar	Southeast Asia	World Bank Lower Middle Income
16	Philippines	Southeast Asia	World Bank Lower Middle Income
17	Sri Lanka	Southeast Asia	World Bank Lower Middle Income
18	Thailand	Southeast Asia	World Bank Upper Middle Income
19	Timor-Leste	Southeast Asia	World Bank Lower Middle Income
20	Vietnam	Southeast Asia	World Bank Lower Middle Income
22	Fiji	Oceania	World Bank Upper Middle Income
23	Kiribati	Oceania	World Bank Lower Middle Income
24	Marshall Islands	Oceania	World Bank Upper Middle Income
25	Federated States of Micronesia	Oceania	World Bank Lower Middle Income
26	Papua New Guinea	Oceania	World Bank Lower Middle Income
27	Samoa	Oceania	World Bank Upper Middle Income
28	Solomon Islands	Oceania	World Bank Lower Middle Income
29	Tonga	Oceania	World Bank Upper Middle Income
30	Vanuatu	Oceania	World Bank Lower Middle Income
33	Armenia	Central Asia	World Bank Lower Middle Income
34	Azerbaijan	Central Asia	World Bank Upper Middle Income
35	Georgia	Central Asia	World Bank Lower Middle Income
36	Kazakhstan	Central Asia	World Bank Upper Middle Income
37	Kyrgyzstan	Central Asia	World Bank Lower Middle Income
38	Mongolia	Central Asia	World Bank Lower Middle Income
39	Tajikistan	Central Asia	World Bank Lower Middle Income
40	Turkmenistan	Central Asia	World Bank Upper Middle Income
41	Uzbekistan	Central Asia	World Bank Lower Middle Income
43	Albania	Central Europe	World Bank Upper Middle Income
44	Bosnia and Herzegovina	Central Europe	World Bank Upper Middle Income
45	Bulgaria	Central Europe	World Bank Upper Middle Income
46	Croatia	Central Europe	World Bank Upper Middle Income
47	Czech Republic	Central Europe	World Bank High Income
48	Hungary	Central Europe	World Bank High Income
49	Macedonia	Central Europe	World Bank Upper Middle Income
50	Montenegro	Central Europe	World Bank Upper Middle Income
51	Poland	Central Europe	World Bank High Income
52	Romania	Central Europe	World Bank Upper Middle Income
53	Serbia	Central Europe	World Bank Upper Middle Income
54	Slovakia	Central Europe	World Bank High Income
55	Slovenia	Central Europe	World Bank High Income
57	Belarus	Eastern Europe	World Bank Upper Middle Income
58	Estonia	Eastern Europe	World Bank High Income
59	Latvia	Eastern Europe	World Bank High Income
60	Lithuania	Eastern Europe	World Bank High Income
61	Moldova	Eastern Europe	World Bank Lower Middle Income
62	Russian Federation	Eastern Europe	World Bank Upper Middle Income
63	Ukraine	Eastern Europe	World Bank Lower Middle Income
66	Brunei	High-income Asia Pacific	World Bank High Income
67	Japan	High-income Asia Pacific	World Bank High Income
68	South Korea	High-income Asia Pacific	World Bank High Income
69	Singapore	High-income Asia Pacific	World Bank High Income
71	Australia	Australasia	World Bank High Income
72	New Zealand	Australasia	World Bank High Income
74	Andorra	Western Europe	World Bank High Income
75	Austria	Western Europe	World Bank High Income

Continuation of Table ??			
Country id	Country name	Region	World Bank group
76	Belgium	Western Europe	World Bank High Income
77	Cyprus	Western Europe	World Bank High Income
78	Denmark	Western Europe	World Bank High Income
79	Finland	Western Europe	World Bank High Income
80	France	Western Europe	World Bank High Income
81	Germany	Western Europe	World Bank High Income
82	Greece	Western Europe	World Bank High Income
83	Iceland	Western Europe	World Bank High Income
84	Ireland	Western Europe	World Bank High Income
85	Israel	Western Europe	World Bank High Income
86	Italy	Western Europe	World Bank High Income
87	Luxembourg	Western Europe	World Bank High Income
88	Malta	Western Europe	World Bank High Income
89	Netherlands	Western Europe	World Bank High Income
90	Norway	Western Europe	World Bank High Income
91	Portugal	Western Europe	World Bank High Income
92	Spain	Western Europe	World Bank High Income
93	Sweden	Western Europe	World Bank High Income
94	Switzerland	Western Europe	World Bank High Income
95	United Kingdom	Western Europe	World Bank High Income
97	Argentina	Southern Latin America	World Bank Upper Middle Income
98	Chile	Southern Latin America	World Bank High Income
99	Uruguay	Southern Latin America	World Bank High Income
101	Canada	High-income North America	World Bank High Income
102	United States	High-income North America	World Bank High Income
105	Antigua and Barbuda	Caribbean	World Bank High Income
106	The Bahamas	Caribbean	World Bank High Income
107	Barbados	Caribbean	World Bank High Income
108	Belize	Caribbean	World Bank Upper Middle Income
109	Cuba	Caribbean	World Bank Upper Middle Income
110	Dominica	Caribbean	World Bank Upper Middle Income
111	Dominican Republic	Caribbean	World Bank Upper Middle Income
112	Grenada	Caribbean	World Bank Upper Middle Income
113	Guyana	Caribbean	World Bank Upper Middle Income
114	Haiti	Caribbean	World Bank Low Income
115	Jamaica	Caribbean	World Bank Upper Middle Income
116	Saint Lucia	Caribbean	World Bank Upper Middle Income
117	Saint Vincent and the Grenadines	Caribbean	World Bank Upper Middle Income
118	Suriname	Caribbean	World Bank Upper Middle Income
119	Trinidad and Tobago	Caribbean	World Bank High Income
121	Bolivia	Andean Latin America	World Bank Lower Middle Income
122	Ecuador	Andean Latin America	World Bank Upper Middle Income
123	Peru	Andean Latin America	World Bank Upper Middle Income
125	Colombia	Central Latin America	World Bank Upper Middle Income
126	Costa Rica	Central Latin America	World Bank Upper Middle Income
127	El Salvador	Central Latin America	World Bank Lower Middle Income
128	Guatemala	Central Latin America	World Bank Lower Middle Income
129	Honduras	Central Latin America	World Bank Lower Middle Income
130	Mexico	Central Latin America	World Bank Upper Middle Income
131	Nicaragua	Central Latin America	World Bank Lower Middle Income
132	Panama	Central Latin America	World Bank Upper Middle Income
133	Venezuela	Central Latin America	World Bank Upper Middle Income
135	Brazil	Tropical Latin America	World Bank Upper Middle Income
136	Paraguay	Tropical Latin America	World Bank Upper Middle Income
139	Algeria	North Africa and Middle East	World Bank Upper Middle Income
140	Bahrain	North Africa and Middle East	World Bank High Income
141	Egypt	North Africa and Middle East	World Bank Lower Middle Income
142	Iran	North Africa and Middle East	World Bank Upper Middle Income
143	Iraq	North Africa and Middle East	World Bank Upper Middle Income
144	Jordan	North Africa and Middle East	World Bank Lower Middle Income

Continuation of Table ??			
Country id	Country name	Region	World Bank group
145	Kuwait	North Africa and Middle East	World Bank High Income
146	Lebanon	North Africa and Middle East	World Bank Upper Middle Income
147	Libya	North Africa and Middle East	World Bank Upper Middle Income
148	Morocco	North Africa and Middle East	World Bank Lower Middle Income
149	Palestine	North Africa and Middle East	World Bank Lower Middle Income
150	Oman	North Africa and Middle East	World Bank High Income
151	Qatar	North Africa and Middle East	World Bank High Income
152	Saudi Arabia	North Africa and Middle East	World Bank High Income
153	Syria	North Africa and Middle East	World Bank Lower Middle Income
154	Tunisia	North Africa and Middle East	World Bank Lower Middle Income
155	Turkey	North Africa and Middle East	World Bank Upper Middle Income
156	United Arab Emirates	North Africa and Middle East	World Bank High Income
157	Yemen	North Africa and Middle East	World Bank Lower Middle Income
160	Afghanistan	North Africa and Middle East	World Bank Low Income
161	Bangladesh	South Asia	World Bank Lower Middle Income
162	Bhutan	South Asia	World Bank Lower Middle Income
163	India	South Asia	World Bank Lower Middle Income
164	Nepal	South Asia	World Bank Low Income
165	Pakistan	South Asia	World Bank Lower Middle Income
168	Angola	Central Sub-Saharan Africa	World Bank Lower Middle Income
169	Central African Republic	Central Sub-Saharan Africa	World Bank Low Income
170	Congo	Central Sub-Saharan Africa	World Bank Lower Middle Income
171	Democratic Republic of the Congo	Central Sub-Saharan Africa	World Bank Low Income
172	Equatorial Guinea	Central Sub-Saharan Africa	World Bank Upper Middle Income
173	Gabon	Central Sub-Saharan Africa	World Bank Upper Middle Income
175	Burundi	Eastern Sub-Saharan Africa	World Bank Low Income
176	Comoros	Eastern Sub-Saharan Africa	World Bank Low Income
177	Djibouti	Eastern Sub-Saharan Africa	World Bank Lower Middle Income
178	Eritrea	Eastern Sub-Saharan Africa	World Bank Low Income
179	Ethiopia	Eastern Sub-Saharan Africa	World Bank Low Income
180	Kenya	Eastern Sub-Saharan Africa	World Bank Lower Middle Income
181	Madagascar	Eastern Sub-Saharan Africa	World Bank Low Income
182	Malawi	Eastern Sub-Saharan Africa	World Bank Low Income
183	Mauritius	Southeast Asia	World Bank Upper Middle Income
184	Mozambique	Eastern Sub-Saharan Africa	World Bank Low Income
185	Rwanda	Eastern Sub-Saharan Africa	World Bank Low Income
186	Seychelles	Southeast Asia	World Bank High Income
187	Somalia	Eastern Sub-Saharan Africa	World Bank Low Income
189	Tanzania	Eastern Sub-Saharan Africa	World Bank Low Income
190	Uganda	Eastern Sub-Saharan Africa	World Bank Low Income
191	Zambia	Eastern Sub-Saharan Africa	World Bank Lower Middle Income
193	Botswana	Southern Sub-Saharan Africa	World Bank Upper Middle Income
194	Lesotho	Southern Sub-Saharan Africa	World Bank Lower Middle Income
195	Namibia	Southern Sub-Saharan Africa	World Bank Upper Middle Income
196	South Africa	Southern Sub-Saharan Africa	World Bank Upper Middle Income
197	Swaziland	Southern Sub-Saharan Africa	World Bank Lower Middle Income
198	Zimbabwe	Southern Sub-Saharan Africa	World Bank Low Income
200	Benin	Western Sub-Saharan Africa	World Bank Low Income
201	Burkina Faso	Western Sub-Saharan Africa	World Bank Low Income
202	Cameroon	Western Sub-Saharan Africa	World Bank Lower Middle Income
203	Cape Verde	Western Sub-Saharan Africa	World Bank Lower Middle Income
204	Chad	Western Sub-Saharan Africa	World Bank Low Income
205	Cote d'Ivoire	Western Sub-Saharan Africa	World Bank Lower Middle Income
206	The Gambia	Western Sub-Saharan Africa	World Bank Low Income
207	Ghana	Western Sub-Saharan Africa	World Bank Lower Middle Income
208	Guinea	Western Sub-Saharan Africa	World Bank Low Income
209	Guinea-Bissau	Western Sub-Saharan Africa	World Bank Low Income
210	Liberia	Western Sub-Saharan Africa	World Bank Low Income
211	Mali	Western Sub-Saharan Africa	World Bank Low Income
212	Mauritania	Western Sub-Saharan Africa	World Bank Lower Middle Income

Continuation of Table ??			
Country id	Country name	Region	World Bank group
213	Niger	Western Sub-Saharan Africa	World Bank Low Income
214	Nigeria	Western Sub-Saharan Africa	World Bank Lower Middle Income
215	Sao Tome and Principe	Western Sub-Saharan Africa	World Bank Lower Middle Income
216	Senegal	Western Sub-Saharan Africa	World Bank Low Income
217	Sierra Leone	Western Sub-Saharan Africa	World Bank Low Income
218	Togo	Western Sub-Saharan Africa	World Bank Low Income
298	American Samoa	Oceania	World Bank Upper Middle Income
305	Bermuda	Caribbean	World Bank High Income
349	Greenland	High-income North America	World Bank High Income
351	Guam	Oceania	World Bank High Income
376	Northern Mariana Islands	Oceania	World Bank High Income
385	Puerto Rico	Caribbean	World Bank High Income
422	Virgin Islands, U.S.	Caribbean	World Bank High Income
435	South Sudan	Eastern Sub-Saharan Africa	World Bank Low Income
522	Sudan	North Africa and Middle East	World Bank Lower Middle Income

Notes: Global Burden of Disease Study 2016
