# Supplementary material for: The development of nations conditions the disease space
Source: PLoS One. 2021 Jan 7;16(1):e0244843. doi: 10.1371/journal.pone.0244843 (PMC7790431; doi:10.1371/journal.pone.0244843)
Supplement: S2 Table — (PDF) [file pone.0244843.s002.pdf]

S2 Table. List of diseases and injuries in the dataset.

Disease id	Disease name	Parent disease	Outline
294	All causes	294	Total
295	Communicable, maternal, neonatal, and nutritional diseases	294	A
296	HIV/AIDS and tuberculosis	295	A.1
297	Tuberculosis	296	A.1.1
934	Drug-susceptible tuberculosis	297	A.1.1.1
946	Multidrug-resistant tuberculosis without extensive drug resistance	297	A.1.1.2
947	Extensively drug-resistant tuberculosis	297	A.1.1.3
954	Latent tuberculosis infection	297	A.1.1.4
298	HIV/AIDS	296	A.1.2
948	Drug-susceptible HIV/AIDS - Tuberculosis	298	A.1.2.1
949	Multidrug-resistant HIV/AIDS - Tuberculosis without extensive drug resistance	298	A.1.2.2
950	Extensively drug-resistant HIV/AIDS - Tuberculosis	298	A.1.2.3
300	HIV/AIDS resulting in other diseases	298	A.1.2.4
301	Diarrhea, lower respiratory, and other common infectious diseases	295	A.2
302	Diarrheal diseases	301	A.2.1
318	Intestinal infectious diseases	301	A.2.2
319	Typhoid fever	318	A.2.2.1
320	Paratyphoid fever	318	A.2.2.2
321	Other intestinal infectious diseases	318	A.2.2.3
322	Lower respiratory infections	301	A.2.3
328	Upper respiratory infections	301	A.2.4
329	Otitis media	301	A.2.5
332	Meningitis	301	A.2.6
333	Pneumococcal meningitis	332	A.2.6.1
334	H influenzae type B meningitis	332	A.2.6.2
335	Meningococcal meningitis	332	A.2.6.3
336	Other meningitis	332	A.2.6.4
337	Encephalitis	301	A.2.7
338	Diphtheria	301	A.2.8
339	Whooping cough	301	A.2.9
340	Tetanus	301	A.2.10
341	Measles	301	A.2.11
342	Varicella and herpes zoster	301	A.2.12

Continuation of Table ??			
Disease id	Disease name	Parent disease	Outline
344	Neglected tropical diseases and malaria	295	A.3
345	Malaria	344	A.3.1
346	Chagas disease	344	A.3.2
347	Leishmaniasis	344	A.3.3
348	Visceral leishmaniasis	347	A.3.3.1
349	Cutaneous and mucocutaneous leishmaniasis	347	A.3.3.2
350	African trypanosomiasis	344	A.3.4
351	Schistosomiasis	344	A.3.5
352	Cysticercosis	344	A.3.6
353	Cystic echinococcosis	344	A.3.7
354	Lymphatic filariasis	344	A.3.8
355	Onchocerciasis	344	A.3.9
356	Trachoma	344	A.3.10
357	Dengue	344	A.3.11
358	Yellow fever	344	A.3.12
359	Rabies	344	A.3.13
360	Intestinal nematode infections	344	A.3.14
361	Ascariasis	360	A.3.14.1
362	Trichuriasis	360	A.3.14.2
363	Hookworm disease	360	A.3.14.3
364	Food-borne trematodiases	344	A.3.15
405	Leprosy	344	A.3.16
843	Ebola	344	A.3.17
935	Zika virus	344	A.3.18
936	Guinea worm disease	344	A.3.19
365	Other neglected tropical diseases	344	A.3.20
366	Maternal disorders	295	A.4
367	Maternal hemorrhage	366	A.4.1
368	Maternal sepsis and other maternal infections	366	A.4.2
369	Maternal hypertensive disorders	366	A.4.3
370	Maternal obstructed labor and uterine rupture	366	A.4.4
371	Maternal abortion, miscarriage, and ectopic pregnancy	366	A.4.5
375	Indirect maternal deaths	366	A.4.6
376	Late maternal deaths	366	A.4.7
741	Maternal deaths aggravated by HIV/AIDS	366	A.4.8
379	Other maternal disorders	366	A.4.9
380	Neonatal disorders	295	A.5
381	Neonatal preterm birth complications	380	A.5.1
382	Neonatal encephalopathy due to birth asphyxia and trauma	380	A.5.2
383	Neonatal sepsis and other neonatal infections	380	A.5.3
384	Hemolytic disease and other neonatal jaundice	380	A.5.4
385	Other neonatal disorders	380	A.5.5
386	Nutritional deficiencies	295	A.6
387	Protein-energy malnutrition	386	A.6.1
388	Iodine deficiency	386	A.6.2
389	Vitamin A deficiency	386	A.6.3
390	Iron-deficiency anemia	386	A.6.4
391	Other nutritional deficiencies	386	A.6.5
392	Other communicable, maternal, neonatal, and nutritional diseases	295	A.7
393	Sexually transmitted diseases excluding HIV	392	A.7.1
394	Syphilis	393	A.7.1.1
395	Chlamydial infection	393	A.7.1.2
396	Gonococcal infection	393	A.7.1.3
397	Trichomoniasis	393	A.7.1.4
398	Genital herpes	393	A.7.1.5
399	Other sexually transmitted diseases	393	A.7.1.6
400	Hepatitis	392	A.7.2
401	Acute hepatitis A	400	A.7.2.1
402	Hepatitis B	400	A.7.2.2
403	Hepatitis C	400	A.7.2.3

Continuation of Table ??			
Disease id	Disease name	Parent disease	Outline
404	Acute hepatitis E	400	A.7.2.4
408	Other infectious diseases	392	A.7.3
409	Non-communicable diseases	294	B
410	Neoplasms	409	B.1
444	Lip and oral cavity cancer	410	B.1.1
447	Nasopharynx cancer	410	B.1.2
450	Other pharynx cancer	410	B.1.3
411	Esophageal cancer	410	B.1.4
414	Stomach cancer	410	B.1.5
441	Colon and rectum cancer	410	B.1.6
417	Liver cancer	410	B.1.7
418	Liver cancer due to hepatitis B	417	B.1.7.1
419	Liver cancer due to hepatitis C	417	B.1.7.2
420	Liver cancer due to alcohol use	417	B.1.7.3
421	Liver cancer due to other causes	417	B.1.7.4
453	Gallbladder and biliary tract cancer	410	B.1.8
456	Pancreatic cancer	410	B.1.9
423	Larynx cancer	410	B.1.10
426	Tracheal, bronchus, and lung cancer	410	B.1.11
459	Malignant skin melanoma	410	B.1.12
462	Non-melanoma skin cancer	410	B.1.13
849	Non-melanoma skin cancer (squamous-cell carcinoma)	462	B.1.13.1
850	Non-melanoma skin cancer (basal-cell carcinoma)	462	B.1.13.2
429	Breast cancer	410	B.1.14
432	Cervical cancer	410	B.1.15
435	Uterine cancer	410	B.1.16
465	Ovarian cancer	410	B.1.17
438	Prostate cancer	410	B.1.18
468	Testicular cancer	410	B.1.19
471	Kidney cancer	410	B.1.20
474	Bladder cancer	410	B.1.21
477	Brain and nervous system cancer	410	B.1.22
480	Thyroid cancer	410	B.1.23
483	Mesothelioma	410	B.1.24
484	Hodgkin lymphoma	410	B.1.25
485	Non-Hodgkin lymphoma	410	B.1.26
486	Multiple myeloma	410	B.1.27
487	Leukemia	410	B.1.28
845	Acute lymphoid leukemia	487	B.1.28.1
846	Chronic lymphoid leukemia	487	B.1.28.2
847	Acute myeloid leukemia	487	B.1.28.3
848	Chronic myeloid leukemia	487	B.1.28.4
943	Other leukemia	487	B.1.28.5
488	Other neoplasms	410	B.1.29
491	Cardiovascular diseases	409	B.2
492	Rheumatic heart disease	491	B.2.1
493	Ischemic heart disease	491	B.2.2
494	Cerebrovascular disease	491	B.2.3
495	Ischemic stroke	494	B.2.3.1
496	Hemorrhagic stroke	494	B.2.3.2
498	Hypertensive heart disease	491	B.2.4
499	Cardiomyopathy and myocarditis	491	B.2.5
942	Myocarditis	499	B.2.5.1
938	Alcoholic cardiomyopathy	499	B.2.5.2
944	Other cardiomyopathy	499	B.2.5.3
500	Atrial fibrillation and flutter	491	B.2.6
501	Aortic aneurysm	491	B.2.7
502	Peripheral artery disease	491	B.2.8
503	Endocarditis	491	B.2.9
507	Other cardiovascular and circulatory diseases	491	B.2.10

Continuation of Table ??			
Disease id	Disease name	Parent disease	Outline
508	Chronic respiratory diseases	409	B.3
509	Chronic obstructive pulmonary disease	508	B.3.1
510	Pneumoconiosis	508	B.3.2
511	Silicosis	510	B.3.2.1
512	Asbestosis	510	B.3.2.2
513	Coal workers pneumoconiosis	510	B.3.2.3
514	Other pneumoconiosis	510	B.3.2.4
515	Asthma	508	B.3.3
516	Interstitial lung disease and pulmonary sarcoidosis	508	B.3.4
520	Other chronic respiratory diseases	508	B.3.5
521	Cirrhosis and other chronic liver diseases	409	B.4
522	Cirrhosis and other chronic liver diseases due to hepatitis B	521	B.4.1
523	Cirrhosis and other chronic liver diseases due to hepatitis C	521	B.4.2
524	Cirrhosis and other chronic liver diseases due to alcohol use	521	B.4.3
525	Cirrhosis and other chronic liver diseases due to other causes	521	B.4.4
526	Digestive diseases	409	B.5
527	Peptic ulcer disease	526	B.5.1
528	Gastritis and duodenitis	526	B.5.2
529	Appendicitis	526	B.5.3
530	Paralytic ileus and intestinal obstruction	526	B.5.4
531	Inguinal, femoral, and abdominal hernia	526	B.5.5
532	Inflammatory bowel disease	526	B.5.6
533	Vascular intestinal disorders	526	B.5.7
534	Gallbladder and biliary diseases	526	B.5.8
535	Pancreatitis	526	B.5.9
541	Other digestive diseases	526	B.5.10
542	Neurological disorders	409	B.6
543	Alzheimer disease and other dementias	542	B.6.1
544	Parkinson disease	542	B.6.2
545	Epilepsy	542	B.6.3
546	Multiple sclerosis	542	B.6.4
554	Motor neuron disease	542	B.6.5
547	Migraine	542	B.6.6
548	Tension-type headache	542	B.6.7
557	Other neurological disorders	542	B.6.8
558	Mental and substance use disorders	409	B.7
559	Schizophrenia	558	B.7.1
560	Alcohol use disorders	558	B.7.2
561	Drug use disorders	558	B.7.3
562	Opioid use disorders	561	B.7.3.1
563	Cocaine use disorders	561	B.7.3.2
564	Amphetamine use disorders	561	B.7.3.3
565	Cannabis use disorders	561	B.7.3.4
566	Other drug use disorders	561	B.7.3.5
567	Depressive disorders	558	B.7.4
568	Major depressive disorder	567	B.7.4.1
569	Dysthymia	567	B.7.4.2
570	Bipolar disorder	558	B.7.5
571	Anxiety disorders	558	B.7.6
572	Eating disorders	558	B.7.7
573	Anorexia nervosa	572	B.7.7.1
574	Bulimia nervosa	572	B.7.7.2
575	Autistic spectrum disorders	558	B.7.8
576	Autism	575	B.7.8.1
577	Asperger syndrome and other autistic spectrum disorders	575	B.7.8.2
578	Attention-deficit/hyperactivity disorder	558	B.7.9
579	Conduct disorder	558	B.7.10
582	Idiopathic developmental intellectual disability	558	B.7.11
585	Other mental and substance use disorders	558	B.7.12
586	Diabetes, urogenital, blood, and endocrine diseases	409	B.8

Continuation of Table ??			
Disease id	Disease name	Parent disease	Outline
587	Diabetes mellitus	586	B.8.1
588	Acute glomerulonephritis	586	B.8.2
589	Chronic kidney disease	586	B.8.3
590	Chronic kidney disease due to diabetes mellitus	589	B.8.3.1
591	Chronic kidney disease due to hypertension	589	B.8.3.2
592	Chronic kidney disease due to glomerulonephritis	589	B.8.3.3
593	Chronic kidney disease due to other causes	589	B.8.3.4
594	Urinary diseases and male infertility	586	B.8.4
595	Interstitial nephritis and urinary tract infections	594	B.8.4.1
596	Urolithiasis	594	B.8.4.2
597	Benign prostatic hyperplasia	594	B.8.4.3
598	Male infertility	594	B.8.4.4
602	Other urinary diseases	594	B.8.4.5
603	Gynecological diseases	586	B.8.5
604	Uterine fibroids	603	B.8.5.1
605	Polycystic ovarian syndrome	603	B.8.5.2
606	Female infertility	603	B.8.5.3
607	Endometriosis	603	B.8.5.4
608	Genital prolapse	603	B.8.5.5
609	Premenstrual syndrome	603	B.8.5.6
612	Other gynecological diseases	603	B.8.5.7
613	Hemoglobinopathies and hemolytic anemias	586	B.8.6
614	Thalassemias	613	B.8.6.1
837	Thalassemias trait	613	B.8.6.2
615	Sickle cell disorders	613	B.8.6.3
838	Sickle cell trait	613	B.8.6.4
616	G6PD deficiency	613	B.8.6.5
839	G6PD trait	613	B.8.6.6
618	Other hemoglobinopathies and hemolytic anemias	613	B.8.6.7
619	Endocrine, metabolic, blood, and immune disorders	586	B.8.7
626	Musculoskeletal disorders	409	B.9
627	Rheumatoid arthritis	626	B.9.1
628	Osteoarthritis	626	B.9.2
629	Low back and neck pain	626	B.9.3
630	Low back pain	629	B.9.3.1
631	Neck pain	629	B.9.3.2
632	Gout	626	B.9.4
639	Other musculoskeletal disorders	626	B.9.5
640	Other non-communicable diseases	409	B.10
641	Congenital birth defects	640	B.10.1
642	Neural tube defects	641	B.10.1.1
643	Congenital heart anomalies	641	B.10.1.2
644	Orofacial clefts	641	B.10.1.3
645	Down syndrome	641	B.10.1.4
646	Turner syndrome	641	B.10.1.5
647	Klinefelter syndrome	641	B.10.1.6
648	Other chromosomal abnormalities	641	B.10.1.7
649	Congenital musculoskeletal and limb anomalies	641	B.10.1.8
650	Urogenital congenital anomalies	641	B.10.1.9
651	Digestive congenital anomalies	641	B.10.1.10
652	Other congenital birth defects	641	B.10.1.11
653	Skin and subcutaneous diseases	640	B.10.2
654	Dermatitis	653	B.10.2.1
655	Psoriasis	653	B.10.2.2
656	Cellulitis	653	B.10.2.3
657	Pyoderma	653	B.10.2.4
658	Scabies	653	B.10.2.5
659	Fungal skin diseases	653	B.10.2.6
660	Viral skin diseases	653	B.10.2.7
661	Acne vulgaris	653	B.10.2.8

Continuation of Table ??			
Disease id	Disease name	Parent disease	Outline
662	Alopecia areata	653	B.10.2.9
663	Pruritus	653	B.10.2.10
664	Urticaria	653	B.10.2.11
665	Decubitus ulcer	653	B.10.2.12
668	Other skin and subcutaneous diseases	653	B.10.2.13
669	Sense organ diseases	640	B.10.3
670	Glaucoma	669	B.10.3.1
671	Cataract	669	B.10.3.2
672	Macular degeneration	669	B.10.3.3
673	Refraction and accommodation disorders	669	B.10.3.4
674	Age-related and other hearing loss	669	B.10.3.5
675	Other vision loss	669	B.10.3.6
679	Other sense organ diseases	669	B.10.3.7
680	Oral disorders	640	B.10.4
681	Caries of deciduous teeth	680	B.10.4.1
682	Caries of permanent teeth	680	B.10.4.2
683	Periodontal diseases	680	B.10.4.3
684	Edentulism and severe tooth loss	680	B.10.4.4
685	Other oral disorders	680	B.10.4.5
686	Sudden infant death syndrome	640	B.10.5
687	Injuries	294	C
688	Transport injuries	687	C.1
689	Road injuries	688	C.1.1
690	Pedestrian road injuries	689	C.1.1.1
691	Cyclist road injuries	689	C.1.1.2
692	Motorcyclist road injuries	689	C.1.1.3
693	Motor vehicle road injuries	689	C.1.1.4
694	Other road injuries	689	C.1.1.5
695	Other transport injuries	688	C.1.2
696	Unintentional injuries	687	C.2
697	Falls	696	C.2.1
698	Drowning	696	C.2.2
699	Fire, heat, and hot substances	696	C.2.3
700	Poisonings	696	C.2.4
704	Exposure to mechanical forces	696	C.2.5
705	Unintentional firearm injuries	704	C.2.5.1
706	Unintentional suffocation	704	C.2.5.2
707	Other exposure to mechanical forces	704	C.2.5.3
708	Adverse effects of medical treatment	696	C.2.6
709	Animal contact	696	C.2.7
710	Venomous animal contact	709	C.2.7.1
711	Non-venomous animal contact	709	C.2.7.2
712	Foreign body	696	C.2.8
713	Pulmonary aspiration and foreign body in airway	712	C.2.8.1
714	Foreign body in eyes	712	C.2.8.2
715	Foreign body in other body part	712	C.2.8.3
842	Environmental heat and cold exposure	696	C.2.9
716	Other unintentional injuries	696	C.2.10
717	Self-harm and interpersonal violence	687	C.3
718	Self-harm	717	C.3.1
721	Self-harm by firearm	718	C.3.1.1
723	Self-harm by other specified means	718	C.3.1.2
724	Interpersonal violence	717	C.3.2
725	Physical violence by firearm	724	C.3.2.1
726	Physical violence by sharp object	724	C.3.2.2
941	Sexual violence	724	C.3.2.3
727	Physical violence by other means	724	C.3.2.4
728	Forces of nature, conflict and terrorism, and executions and police conflict	687	C.4
729	Exposure to forces of nature	728	C.4.1
945	Conflict and terrorism	728	C.4.3

Continuation of Table ??			
Disease id	Disease name	Parent disease	Outline
854	Executions and police conflict	728	C.4.2
Notes: Global Burden of Disease Study 2016			
