# Supplementary material for: The development of nations conditions the disease space
Source: PLoS One. 2021 Jan 7;16(1):e0244843. doi: 10.1371/journal.pone.0244843 (PMC7790431; doi:10.1371/journal.pone.0244843)
Supplement: S1 Appendix — (PDF) [file pone.0244843.s003.pdf]

S1 Appendix: List of countries in regressions Afghanistan, Albania, Algeria, Angola, Antigua and Barbuda, Argentina, Armenia, Australia, Austria, Azerbaijan, Bahamas, Bahrain, Bangladesh, Barbados, Belarus, Belgium, Belize, Benin, Bhutan, Bolivia, Bosnia and Herzegovina, Botswana, Brazil, Brunei, Darussalam, Bulgaria, Burkina Faso, Burundi, Cabo Verde, Cambodia, Cameroon, Canada, Central African Republic, Chad, Chile, China, Colombia, Comoros, Rep. of the Congo, Costa Rica, Cote d'Ivoire, Croatia, Cyprus, Czech Republic, Denmark, Dominican Republic, Ecuador, Arab Rep. of Egypt, El Salvador, Equatorial Guinea, Estonia, Eswatini, Ethiopia, Fiji, Finland, France, Gabon, Gambia, Georgia, Germany, Ghana, Greece, Grenada, Guatemala, Guinea, Guinea-Bissau, Guyana, Haiti, Honduras, Hungary, Iceland, India, Indonesia, Islamic Rep. of Iran, Iraq, Ireland, Israel, Italy, Jamaica, Japan, Jordan, Kazakhstan, Kenya, Kiribati, Rep. of Korea, Kuwait, Kyrgyz Republic, Lao PDR, Latvia, Lebanon, Lesotho, Liberia, Libya, Lithuania, Luxembourg, Macedonia FYR, Madagascar, Malawi, Malaysia, Maldives, Malta, Mauritania, Mauritius, Mexico, Moldova, Mongolia, Montenegro, Morocco, Mozambique, Myanmar, Namibia, Nepal, Netherlands, New Zealand, Nicaragua, Niger, Nigeria, Norway, Oman, Pakistan, Panama, Papua New Guinea, Paraguay, Peru, Philippines, Poland, Portugal, Qatar, Russian Federation, Rwanda, Samoa, Sao Tome and Principe, Saudi Arabia, Senegal, Serbia, Seychelles, Sierra Leone, Singapore, Slovak Republic, Slovenia, South Africa, Spain, Sri Lanka, St. Lucia, St. Vincent and the Grenadines, Sudan, Suriname, Sweden, Switzerland, Tajikistan, Tanzania, Thailand, Togo, Tonga, Trinidad and Tobago, Tunisia, Turkey, Uganda, Ukraine, United Arab Emirates, United Kingdom, United States, Uruguay, Vanuatu, Venezuela RB, Vietnam, Rep. of Yemen, Zambia, Zimbabwe.
